# Unlocking the health potential of fermented foods: insights from traditional Indian diets

**DOI:** 10.3389/fmicb.2026.1860268

**Published:** 2026-06-29

**Authors:** Rohith Raali, Nandini Sampath, Guruganesan R. S., Tarunteja J. K., Harsh Vardhan J., Janani Shenbagam B., Velugoti Geethanjali, Venkat Kumar S., Sreeja Shanmuga Doss

**Affiliations:** School of BioSciences and Technology, Vellore Institute of Technology, Vellore, Tamil Nadu, India

**Keywords:** anticancer activity, anticholesterolemic activity, antidiabetic activity, antimicrobial activity, Indian fermented food

## Abstract

Fermented foods have been an integral component of human diets for more than 10 millennia and are created through controlled microbial growth and enzymatic actions. Recent research has demonstrated that fermented foods, such as those containing probiotics and bioactive molecules, can help prevent and manage a wide range of diseases, including diabetes, hypercholesterolemia, cancer, infectious diseases, and psychotic illnesses. While the benefits of fermented foods from the Asian continent have been widely accepted based on animal studies and human trials, the potential of Indian fermented foods has not received the attention it deserves. Traditional Indian diets contain a significant portion of fermented foods, and the country’s diverse geography and cultures offer a wide variety of fermented options. There is increasing evidence supporting the health benefits of Indian fermented foods in managing and preventing various diseases. Hence, this review aims to provide an overview of the current literature on health benefits conferred by Indian fermented food in the context of managing chronic diseases such as diabetes, hypercholesterolemia, cancer and infectious diseases along with mechanistic insights behind the probiotic action.

## Graphical abstract

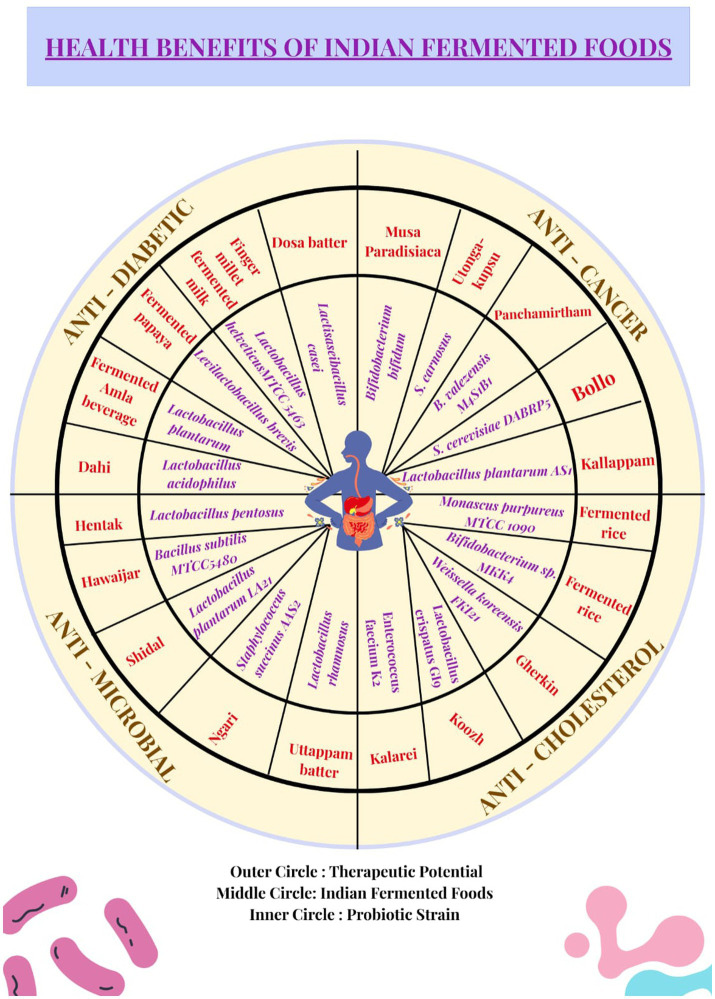

## 1 Introduction

The fast-paced modern lifestyle and changing environmental conditions have led to high prevalence of diseases such as diabetes, hypercholesterolemia, psychological ailments, inflammatory diseases, and cancer among mankind (Garg, 2025; Macia et al., 2021). Although medical treatment for many of these ailments have been successfully developed, recent research has shown a new route to manage and prevent these ailments through improving gut health (Aziz et al., 2024). Gut harbors several millions of microorganisms which are referred to as the gut microbiota. Gut health refers to preserving the right proportion of different classes of beneficial bacteria in the gut (Van Hul et al., 2024). The gut microbiota plays a crucial role in improving the overall health of the body by performing several important functions in the gut. Major beneficial functions of the gut microbiota include breaking down complex nutrients into easily assimilated nutrients, production of bacteriocins that eliminate pathogenic microorganisms from the gut (Biswas et al., 2017), production of enzymes that neutralize reactive oxygen species (delCarmen et al., 2017), production of enzymes involved in cholesterol metabolism, production of metabolites capable of alteration of expression of genes involved in inflammation and cell proliferation (Devi et al., 2018; Miraghajani et al., 2017), stimulation of immune system, production of neurotransmitters (Kanklai et al., 2021), regulating the expression of gut hormones (Sharma et al., 2021), etc. Hence, a healthy gut has been correlated with better management of many of the above-mentioned diseases.

To improve gut health, incorporating fermented foods that are rich in beneficial bacteria into one’s diet has been recommended (Chilton et al., 2015). When we look into our history, health enhancing fermented foods have played a crucial role in sustaining and supporting human societies worldwide (Tamang et al., 2020). Fermentation is a metabolic process by which microorganisms derive energy through the biochemical conversion of organic compounds, particularly carbohydrates, into simpler metabolites such as alcohols, organic acids, and gases under anaerobic or oxygen-limited conditions (Chilton et al., 2015). Although in the earlier days, fermentation was used as a mode of preserving food from contaminating microorganisms, recent research shows that the nutrients released after microbial fermentation can be classified as `naturally fortified functional nutrients’ as they improve overall body health (Alhodieb, 2026). Fermentation also increases bioavailability of phenolic compounds which are potential antioxidants.

The beneficial bacteria present in fermented food are termed as probiotics. World Health Organization (WHO) defines probiotics as ‘live microorganisms which when administered in adequate amounts confer a health benefit on the host’ (Kalantzopoulos, 1997). According to the guidelines for evaluating probiotics put forth by the Food and Agriculture Organization and the WHO, a bacterium is categorized as probiotic only if it possesses the following characteristics in addition to conferring a health benefit: high bile salt tolerance and high acid tolerance to withstand the conditions in the stomach; high auto aggregation and co-aggregation ability that promotes formation of clumps of similar and genetically distinct bacteria which aid in the formation of an intestinal barrier; high adherence to epithelial cells which prevent intestinal colonization of pathogenic microorganisms; absence of haemolytic activity that ensures that blood cells are not degraded; sensitivity to clinically important antibiotics; and antibacterial activity against potential pathogens (Ayyash et al., 2021; Byakika et al., 2019). The most common probiotic family of bacteria in fermented foods is lactic acid bacteria (LAB) which are gram positive, rod-shaped and catalase negative family of bacteria. *Lactobacillus* and *Bifidobacterium* are the two major genuses of LAB found in fermented food that confer a variety of health benefits. *Levilactobacillus brevis, Lactiplantibacillus plantarum, Lactiplantibacillus pentosus, Lacticaseibacillus paracasei, Lacticaseibacillus rhamnosus, limosilactobacillus reuteri* and *Lacticaseibacillus casei* are some prominent *Lactobacillus* species found in fermented food. Similarly, some common *Bifidobacterium* species include *Bifidobacterium adolescentis, Bifidobacterium lactis and Bifidobacterium longum*. In addition to these two genuses, *Enterococcus, Pediococcus, Lueconostoc, Streptococci and Weisella* are other LABs that have gained importance due to their probiotic potential (Hill et al., 2014; Holzapfel et al., 2001).

Several probiotics isolated from Indian fermented foods have been evaluated for their ameliorative effects against diseases like hypercholesterolemia, diabetes, cancer, mental illnesses, and pathogenic infections, etc. *Lactobacillus paraplantarum* isolated from ‘gundruk’ and *Lactobacillus plantarum* MTCC 5422 isolated from ‘fermented cereal’ was found to exhibit anti-inflammatory activity (Devi et al., 2018). The antidiabetic activity of probiotic *‘*dahi*’* containing *Lactobacillus acidophilus* and *Lactobacillus casei* was demonstrated in high fructose fed rats (Yadav et al., 2007). *Lactobacillus fermentum* was isolated from traditional fermented food and its anti-cholesterol activity was demonstrated in wistar rats (Thakkar et al., 2020).

There is a wide acceptance of the health benefits of fermented food from Asian origin such as natto (Japan), miso (Japan), amazake (Japan), kimchi (South Korea) and tempeh (Indonesia and Malaysia) based on animal studies and human trials (Santa et al., 2025). However, there is less emphasis on the therapeutic effects of Indian fermented food (Samantaray and Saha, 2026; Santa et al., 2025). Hence, the current article attempts to summarize the evidence-based research on the therapeutic potential of Indian fermented food to manage several diseases such as cancer, diabetes, hypercholesterolemia, and microbial infections. It throws light on how these foods confer therapeutic effects and suggests commercialization of indigenous probiotic strains. The article concludes with pointers for future research to realize the full potential of Indian fermented foods in treating maladies.

## 2 Traditional Indian fermented foods: substrates and probiotic composition

India has a large diversity of fermented food that have their origins in different parts of the country. ‘Apong’ is a rice based fermented beverage, commonly consumed in north-eastern parts of India (Ray et al., 2016). *‘*Idli*’, ‘*dosa*’, ‘*dhokla*’, ‘*uttapam*’* are staple fermented foods in India that are prepared using combinations of cereals (e.g., rice) and legumes (e.g., black gram or chickpea flour), and are therefore classified as cereal-legume-based fermented products (Ray et al., 2016). A finger millet-based fermented product, ‘ambali’, that contains high calcium and low resistant starch is consumed in southern states of India like Karnataka and Tamil Nadu (Sarkar et al., 2015). *‘*Kambu koozh*’* and *‘*ragi koozh*’*, both are non-alcoholic and naturally fermented foods, consumed as a traditional beverage in southern rural parts of India (Anandharaj et al., 2015). North-eastern states of India contain a variety of soybean fermented foods which includes *‘*kinema*’, ‘*hawaijar*’* and *‘*akhuni*’* (Chaudhary et al., 2018).

There is sufficient evidence where probiotic strains are derived from these Indian fermented foods. For example, *Weissella cibaria* and *Enterococcus lactis* were isolated from *‘*ragi koozh*’* and *‘*jalebi batter*’* demonstrated important probiotic traits such as tolerance to gastrointestinal conditions and antimicrobial activity (Manovina et al., 2022). In another study, *Levilactobacillus brevis* MYSN105 was isolated from fermented *‘*pozha*’* and was found to possess probiotic and antifungal properties (Somashekaraiah et al., 2021). *Weissella confusa* strain GCC_19R1 was isolated from ‘fermented sour rice’, showed key probiotic attributes such as auto aggregation capability, tolerance to gastric juice, antagonistic activity against other pathogens, and absence of haemolytic activity, thus supporting its potential application as a functional starter culture (Nath et al., 2021). A comprehensive list of various Indian fermented foods, the substrates used in their preparation, the probiotics isolated from these foods, and their corresponding geographical indications is provided in Table 1.

**Table 1:** Indian fermented foods and their associated substrates and probiotic microorganisms.

Fermented food	Substrate	Probiotic microbe	Geographical location	References
Panchamirtham	Fruit Mixture	*Bacillus valezensis* M4S1B1, *Proteus terrae* M7S2B1 313	South India	Uma Maheshwari et al. (2019)
Tomato Pickle	Tomato	*Weissella cibaria* p3B	Pan India	Ankaiah et al. (2021)
	Tomato	*Pediococcus acidilactici* TMAB26		Arun et al. (2019)
Utonga-kupsu	Fish	*Staphylococcus carnosus*, *Staphylococcus piscifermentans*	North East India	Singh et al. (2018)
Dasamoolarishta	Dashamula (ten roots)	*Bacillus licheniformis* AG-06, *Bacillus albus* DM-15	South India	Vinothkanna et al. (2022)
Kallappam	Rice	*Lactobacillus plantarum* AS1	South India	Barigela and Bhukya (2021)
Fermented banana	Banana	*Lactobacillus casei*, *Bifidobacterium bifidum*	Pan India	Satish Kumar et al. (2012)
Dahi	Milk	*Lactobacillus acidophilus*, *Bifidobacterium bifidum*	Pan India	Mohania et al. (2013)
	Milk	*Lactobacillus acidophilus*, *Lactobacillus lactis*, *Lactobacillus casei*		Yadav et al. (2007)
	Milk	*Lactobacillus fermentum* strains, i.e., PH5 (handva batter isolate) and PD2 (dosa batter isolate).		Thakkar et al. (2020)
Babru	Black Gram dal	*Lactobacillus plantarum* strains (AdF3, AdF5, AdF6, AdF7, AdF9, AdF10), *Enterococcus faecium* strains (AdF11, AdF2), *Saccharomyces cerevisiae* strains (Sc12, Sc04, Sc17)	North India	Walia et al. (2014)
Fermented rice	Rice	*Bifidobacterium* sp., *Lactobacillus* sp.	Pan India	Hor et al. (2022)
	Rice	Bifidobacterium sp. MKK4		Ray et al. (2018)
Milk-millet composite	Finger millet	*Lactobacillus helveticus* MTCC 5463	Pan India	Chaudhary and Mudgal (2020a)
Millet-legume based Indian traditional fermented product	Millet and legume	*Lactobacillus plantarum*, *Lactiplantibacillus plantarum*, *Enterococcus faecalis, Saccharomyces cerevisiae*	Pan India	Devi and Rajendran (2023)
Sprouted and fermented quinoa	Quinoa	Not mentioned	Pan India	de Lopes et al. (2019)
Fermented amla beverage	Gooseberry	*Pediococcus lolii, Lactobacillus plantarum, Pediococcus acidilactici, Pediococcus pennaceous*	Pan India	Modi et al. (2023)
Fermented papaya	Papaya	*Levilactobacillus brevis* RAMULAB52	Pan India	Sreepathi et al. (2023)
Skim Milk and Dahi (Yogurt)	Milk	*Lactococcus lactis* ssp. *lactis*, *L. lactis* ssp. *cremoris*, *L. lactis* ssp. *diacetylactis*, *Leuconostoc citrovorum*	Pan India	Yadav et al. (2006)
Dosa batter	Rice and lentil	*Limosilactobacillus fermentum, Lacticaseibacillus casei*	Pan India	Chandana Kumari et al. (2022)
	Rice	*Lactobacillus plantarum* LD4		Kumar et al. (2016)
Fermented buffalo and camel Milk	Buffalo and camel milk	*Limosilactobacillus fermentum* (KGL4), *Saccharomyces cerevisiae* (WBS2A)	Pan India	Khakhariya et al. (2023)
Fermented jalebi, medhu vada, and kallappam batters	Rice/wheat based fermented mixtures	*Lacticaseibacillus rhamnosus* RAMULAB13, *Lactiplantibacillus plantarum* RAMULAB14, *Lactiplantibacillus pentosus* RAMULAB15, *Lacticaseibacillus paracasei* RAMULAB16, *Lacticaseibacillus casei* RAMULAB17, *Lacticaseibacillus casei* RAMULAB20, and *Lacticaseibacillus paracasei* RAMULAB21	South India	Huligere et al. (2023)
Fermented koozh (rice based food) and gherkin (fermented cucumber)	Ragi and cucumber	*Weissella koreensis* FKI21, *Lactobacillus crispatus* GI9	South India	Anandharaj et al. (2015)
Kalarei (Cheese)	Milk	*Enterococcus faecium* K2	North India	Bhat et al. (2019)
Yogurt	Milk	*Enterococcus faecium* Chloe1, *Enterococcus faecium* EF, *Lactobacillus pentosus* 7MP	Pan India	Walhe et al. (2021)
	Milk	*Bifidobacterium bifidum* 2,715		Athrayilkkalathil and Prasad (1997)
Idli, Dhokla	Idli, Dhokla	*Pediococcus pentosaceus* G11, *Pediococcus pentosaceus* AP3	South India, West India	Rajan et al. (2021)
Fermented cereal-pulse food mixture	Cereal and pulse	*Lactobacillus casei* (NCDC 19), *Saccharomyces boulardii*	Pan India	Sindhu and Khetarpaul (2003)
Fermented milk	Milk	*Lactobacillus rhamnosus* 5,957, *Lactobacillus rhamnosus* 5,897	Pan India	Yadav et al. (2019)
Tungtap and Tungrymbai	Fish and Soybean	*Lactobacillus plantarum, Lactobacillus brevis*, *Lactobacillus casei*, *Lactobacillus fermentum*, *Pediococcus pentosaceus*, *Enterococcus* sp.	North east India	Biswas et al. (2017)
Uttappam batter	Rice	*Lactobacillus rhamnosus, Lactobacillus plantarum, Lactobacillus brevis*	South India	Jeevaratnam et al. (2015)
Hentak	Fish	*Lactobacillus pentosus*	South India	Aarti et al. (2016)
Kinema	Soybean	*Lactobacillus plantarum* DHCU70, *Lactobacillus plantarum* DKP1	North east India	Goel et al. (2020)
Shidal	Fish	*Lactobacillus plantarum* LA21	North east India	Leslie et al. (2021)

## 3 Anticancer activity of Indian fermented foods

The WHO describes cancer as a group of disease characterized by uncontrolled cell proliferation that can emerge in any part of the body and can later invade other organs in the body (World Health Organization, 2026a). The abnormal cell proliferation is attributed to genetic mutations which can be caused by several factors such as physical (ultraviolet and ionizing radiation), chemical (asbestos, tobacco smoke, alcohol, arsenic) and biological carcinogens (certain viruses or bacteria) (Hassanpour and Dehghani, 2017) Cancer is the first or the second leading cause of death in 118 countries and has caused 10 million deaths worldwide in 2020 (Sung et al., 2021). Cancer leads to severe systemic consequences including tissue invasion, metastasis, chronic inflammation, immune dysregulation, cachexia, organ dysfunction, and reduced quality of life, which collectively contribute to high morbidity and mortality (Hanahan and Weinberg, 2011; Fearon et al., 2011; Mantovani et al., 2008).

Cancer is often regarded as a multifactorial disease, influenced by genetic, environmental, and lifestyle factors, and sometimes attributed to the gut microbiota (Ames and Gold, 1997; Sheflin et al., 2014). Hence, supplementation of fermented foods in one’s diet could act as a prophylactic and prevent or at best act in tandem with prescribed medication to effectively paralyze carcinogenesis. Several Indian fermented foods containing probiotics have been reported to display anticancer properties through a plethora of mechanisms such as antiproliferative activity (Ankaiah et al., 2021; Uma Maheshwari et al., 2019), cytotoxicity (Ankaiah et al., 2021; Barigela and Bhukya, 2021; Singh et al., 2018; Satish Kumar et al., 2012; Vinothkanna et al., 2022), antioxidant activity (Satish Kumar et al., 2012), anti-inflammatory activity (Barigela and Bhukya, 2021), immunomodulation (Mohania et al., 2013), epigenetic modification (Arun et al., 2019), inhibition of *β*-glucuronidase (Arun et al., 2019), and metabolic transformation of carcinogens and antigenotoxicity (Walia et al., 2014) based on *in vitro* and *in vivo* studies. As shown in Table 2, among these modes of action, epigenetic modification and inhibition of β-glucuronidase (Arun et al., 2019) have been attributed to probiotic-derived metabolites while the other mechanisms are considered to be due to the probiotic strains. The anticancer activity of Indian fermented food with evidence from literature is elaborated in the following sections in accordance with their mechanism of action. Table 3 provides a summary of the key findings of studies on Indian fermented food with anticancer potential. Supplementary Table S1 elaborates on the methodology adopted in each of the studies in detail and reiterates the key findings of these studies.

**Table 2:** Mechanism of action of probiotic containing Indian fermented food.

Health benefit of probiotic containing Indian fermented food	Putative mechanism of action attributed to probiotics	Probiotic-derived metabolites and its putative mechanism of action
Anticancer activity	Antiproliferative activity (Ankaiah et al., 2021; Uma Maheshwari et al., 2019) Cytotoxicity (Ankaiah et al., 2021; Barigela and Bhukya, 2021; Singh et al., 2018; Satish Kumar et al., 2012; Vinothkanna et al., 2022) Antioxidant activity (Satish Kumar et al., 2012) Anti-inflammatory activity (Barigela and Bhukya, 2021) Immunomodulation (Mohania et al., 2013) Metabolic transformation of carcinogens (Walia et al., 2014)	Short chain fatty acids (SCFA)-epigenetic modification leading upregulation of apoptosis in cancer cells (Arun et al., 2019) Cell free supernatant containing uncharacterized metabolite-inhibition of *β*-glucuronidase (Arun et al., 2019)
Antidiabetic activity	Increased insulin sensitivity by improving GLUT4 glucose transporter expression in liver tissue (Hor et al., 2022) Reduced insulin secretion (Hor et al., 2022; Sreepathi et al., 2023)	Polyphenols- inhibition of *α*-amylase and α-glucosidase (Chaudhary and Mudgal, 2020a) Dietary fiber- inhibition of α-amylase and α-glucosidase (Chaudhary and Mudgal, 2020a) Hydroxycitric acid- antioxidant activity (Sreepathi et al., 2023) Bioactive peptides (10–75 KDa)- inhibition of α-amylase and α-glucosidase (Khakhariya et al., 2023) Dietary fiber-reduced adipogenesis (de Lopes et al., 2019)
Antihypercholesterolemic activity	Bile salt hydrolase activity (Anandharaj et al., 2015; Athrayilkkalathil and Prasad, 1997; Ray et al., 2018; Rajan et al., 2021; Walhe et al., 2021) Cholesterol assimilation or adsorption (Anandharaj et al., 2015; Bhat et al., 2019). Liver protective effects by improving the activity of ROS scavenging enzymes in the liver (Yadav et al., 2019).	NA
Antibacterial activity	NA	Bacteriocin- cell lysis through pore formation in the bacterial cell wall (Goel et al., 2020; Kumar et al., 2016) Bacteriocin-like inhibitory substance (BLIS)- Unknown mechanism of action (Leslie et al., 2021) 2-Hydroxyl Indole-3-Propanamide- Unknown mechanism of action (Jeevaratnam et al., 2015)

**Table 3:** Summary of key highlights of studies on anticancer effects of various Indian fermented foods and their probiotic strains.

S. No	Indian fermented food	Probiotic strain	Methodology of the study	Antcancer activity reported	References
1.	Panchamirtham (A fermented fruit mix made from banana, brown sugar, seedless dates, sugar candy, honey, cardamom and ghee)	*Bacillus valezensis* M4S1B1, *Proteus terrae* M7S2B1 313	*In vitro* tests using A549 cell line	Anti-proliferative activity	Uma Maheshwari et al. (2019)
2.	Tomato Pickle	*Weissella cibaria* p3B	*In vitro* study using HeLa cell line	Anti-proliferative activity	Ankaiah et al. (2021)
3.	Utonga-kupsu (Manipuri fermented fish product)	*Staphylococcus carnosus, Staphylococcus piscifermentans*	*In vitro* study using HeLa, HT-29 and normal lung cell lines	Cytotoxicity	Singh et al. (2018)
4.	Dasamoolarishta (Indian Ayurvedic fermented traditional medicine)	*Bacillus licheniformis* AG-06, *Bacillus albus* DM-15	*In-vitro* study using A549 cancer cell line	Cytotoxicity	Vinothkanna et al. (2022)
5.	Tomato Pickle	*Pediococcus acidilactici* TMAB26	*In vitro* study using HT-29 and Caco-2 cell lines	Cytotoxicity, Anti-inflammatory	Barigela and Bhukya (2021)
6.	Kallappam (South Indian fermented food made with rice flour and coconut batter)	*Lactobacillus plantarum* AS1	*In vivo* study using normal and tumor induced rats	Antioxidant activity, cytotoxicity	Satish Kumar et al. (2012)
7.	Fermented banana (*Musa Paradisiaca*)	*Lactobacillus casei, Bifidobacterium bifidum*	*In vitro* study using HT29 cell lines	Cytotoxicity, production of SCFA, inhibition of β-glucuronidase	Arun et al. (2019)
8.	Dahi (Indian fermented milk product)	*Lactobacillus acidophilus, Bifidobacterium bifidum*	*In vivo* study involving normal rats and cancer induced rats	Immunomodulation	Mohania et al. (2013)
9.	Babru (Pancakes made from fermented batter), Aara, Apple wine (Fermented drinks of North-Western Himalayas)	*Lactobacillus plantarum* strains (AdF3, AdF5, AdF6, AdF7, AdF9, AdF10), *Enterococcus faecium* strains (AdF11, AdF2), *Saccharomyces cerevisiae* strains (Sc12, Sc04, Sc17)	*In vitro* study where the isolated strains were assessed for their ability to inhibit genotoxins and mutagens through SOS chromotest and Ames test. Viability of the strains against 4-NQO and furazolidone, and their ability to bind and transform carcinogens were also evaluated.	Metabolic transformation of carcinogens, antigenotoxicity	Walia et al. (2014)

‘Panchamirtham’ is an Indian traditional religious offering made with fermented mixtures of banana, jaggery, dates, cane sugar, honey, cardamom, and clarified butter. 16S rRNA gene sequencing by Uma Maheshwari et al. (2019) revealed the presence of over 100 species of bacteria with strong anticancer activity against A549 cancer cell line by the virtue of reducing proliferation, causing cellular shrinkage and membrane damage. In specific, *Bacillus valezensis* M4S1B1 and *Proteus terrae* M7S2B1 313, showed potential activity against lung cancer cell line A549 with low IC_50_ values of 402 ± 7 μg/mL and 417 ± 2 μg/mL, respectively (Uma Maheshwari et al., 2019). Similarly, the probiotic strain *Weissella cibaria* p3b isolated from ‘Indian tomato pickle’ was found to display anti-proliferative activity against HeLa cells *in vitro* upto 50–70% (Ankaiah et al., 2021). However, the signaling cascade through which the probiotic imparts anti-proliferative activity is not clearly understood.

Numerous probiotic strains isolated from Indian fermented food have also shown cytotoxicity against various cancer cell lines during *in vitro* studies. For example, *in vitro* studies revealed the cytotoxic effect of probiotic strains isolated from ‘Utonga-kupsu’, a Manipuri delicacy. *Staphylococcus carnosus* and *Staphylococcus piscifermentans* present in ‘Utonga-kupsu’ displayed a high cytotoxicity upto 78% against HT-29 and HeLa cell lines. Interestingly, when the probiotic strains were tested against normal lung cell line, a low cytotoxicity of 12% was observed, demonstrating the specificity of cytotoxicity against tumor cells over normal cells (Singh et al., 2018). Probiotic strains isolated from ‘Indian Fermented banana’, viz., *Lactobacillus casei and Bifidobacterium bifidum*, also demonstrated cytotoxicity toward cancer cell lines (Arun et al., 2019). In another study, probiotic strain, *Pediococcus acidilactici* TMAB26 isolated from ‘Indian tomato pickle’ exhibited 94.91% cytotoxic activity against intestinal cancer cells and 92.63% against Caco-2 cancer cells (Barigela and Bhukya, 2021). In addition, when *Bacillus licheniformis* AG-06 and *Bacillus albus* DM-15 isolated from ‘Dasamoolarishta’ (Ayurvedic concoction) were tested against A549 cancer cell lines, the strains exhibited antiproliferative activity, with significant cytotoxicity observed at 20 μg/mL (Vinothkanna et al., 2022). Deciphering the signaling pathway through which probiotic strains confer cytotoxicity can help in the effective development of therapeutic applications.

Indian fermented food rich in antioxidants, that are natural neutralizers of ROS, have shown to stun tumor growth and proliferation. During in *in-vivo* studies, where rats with 1,2-dimethylhydrazine induced colon cancer were orally administered *Lactobacillus plantarum* AS1 isolated from ‘Kalappam’ (South Indian fermented food), a 42.13% reduction in tumor number and a 36.12% reduction in tumor size compared to control was observed. During *in vitro* studies *L. plantarum* AS1 demonstrated 50.96% inhibition of linoleic acid peroxidation and 29.15% free radical scavenging activity. Hence, the potent antioxidant activity of *Lactobacillus plantarum* AS1 is considered to be one of the contributing factors underlying its observed anticancer activity (Satish Kumar et al., 2012). Future work must focus on identification of specific antioxidants present in the fermented foods which can pave way for efficient translation of the anticancer property into clinical settings.

In addition, probiotics have also shown to possess immunomodulatory capabilities, with abilities to reverse immune evasion by cancer cells. ‘Dahi’ (Indian curd- a fermented milk product) containing *Lactobacillus acidophilus* and *Bifidobacterium bifidum* was fed to male wistar mice with 1,2-dimethylhydrazine induced colorectal cancer. The study reported a lower expression of PD-1 (Programmed cell death protein-1) on the T cell surface which promoted infiltration of T cells into the tumor. This demonstrates the anticancer activity of ‘dahi’ through immunomodulation (Mohania et al., 2013).

Chronic inflammation is one of the hallmarks of cancer and hence anti-inflammation is a potential way to mitigate cancer. Probiotic stains such as *Pediococcus acidilactici* isolated from fermented ‘Indian tomato pickle’ has shown to upregulate IL-10, an anti-inflammatory cytokine, and concurrently downregulate proinflammatory markers such as TNF- *α* and IL-6 by 3- and 8-fold, respectively, during *in vitro* studies using HT-29 and Caco-2 cell lines, thereby clearly demonstrating the anti-tumorigenic capability of the isolated probiotic strains (Barigela and Bhukya, 2021).

Metabolites such as short chain fatty acids (SCFAs) present in fermented food have been shown to act as histone deacetylase inhibitors which can cause epigenetic changes leading to enhanced tumor suppressor gene activity, activation of the apoptosis cascade and increased p21 levels to induce cell cycle arrest, thereby reducing the growth of certain tumors (Licciardi et al., 2011). ‘Indian Fermented banana’ (*Musa Paradisiaca*) was found to contain significant levels of SCFAs and has been demonstrated to improve apoptosis of HT29 colon cancer cells (Arun et al., 2019).

Another route through which anticancer activity is established is by the inhibition of *β*-glucuronidase, a key enzyme that prevents excretion of carcinogenic compounds from the body and reactivates them in the colon. Probiotic strains, *Lactobacillus casei and Bifidobacterium bifidum,* were isolated from ‘Indian Fermented banana’ (*Musa Paradisiaca*). During *in-vitro* studies, cell-free supernatants of *L. casei and B. bifidum* showed 42.36 and 53.78% inhibition of β-glucuronidase activity, indicating the capability of the ‘Indian Fermented banana’ in eliminating carcinogens from the body and its potential use as a prophylactic dietary intervention (Arun et al., 2019).

Finally, recent discoveries reveal that probiotic microorganism act as DNA bio-protective agents by quenching mutagens and other DNA reactive compounds. Strains of *Lactobacillus plantarum* and *Saccharomyces cerevisiae* isolated from ‘Aara’, ‘Babru’ and ‘Apple wine’ (common northern Himalayan fermented beverages) were shown to possess antigenotoxic and antimutagenic properties. The strains displayed high antimutagenicity against tested genotoxins (70%), high antigenotoxicity with over 90% inhibition of 4-NQO and 75% inhibition of furazolidone. The strains were shown to effectively bind and metabolically transform the tested carcinogens (Walia et al., 2014).

Hence, Indian fermented foods confer various anticancer activity and hold significant potential in the development of therapeutic intervention for cancer. Validation of the findings using randomized controlled human trials should be the prime focus of future research along with obtaining a comprehensive understanding of the signaling cascades through which probiotics exert their activity.

## 4 Antidiabetic activity of Indian fermented foods

Diabetes mellitus is a metabolic disorder characterized by hyperglycaemia due to lack of insulin secretion or insulin action or both (American Diabetes Association, 2010). There are two types of diabetes mellitus. Type 1 diabetes mellitus is characterized by deficiency in insulin secretion due to autoimmune destruction pancreatic beta cells. On the other hand, type 2 diabetes mellitus is defined by insulin resistance dominantly caused due to obesity, stress and advancing age (American Diabetes Association, 2010). According to WHO, diabetes caused 2 million deaths worldwide in 2021 and the number of people diagnosed with diabetes has quadrupled in the last three decades (World Health Organization, 2024). Prolonged diabetes can lead to severe health complications like kidney failure, heart attack, stroke, loss of vision, and neuropathy leading to lower limb amputation (American Diabetes Association, 2010; World Health Organization, 2024).

Indian fermented foods have shown promising antidiabetic effects through *in vitro* and *in vivo* studies demonstrating their ability to mitigate diabetes-related symptoms. Through *in vivo* studies using animal models, several studies have reported the ability of probiotics present in Indian fermented food to mitigate symptoms of diabetes such as polyphagia, polydipsia, high glucose levels (Chaudhary and Mudgal, 2020a, 2020b; Devi and Rajendran, 2023; Jeong et al., 2021; Tsao et al., 1999). Studies also have shown that fermented food can further decrease the complications arising from diabetes such as nephropathy, abnormal lipid metabolism and abnormal liver function (Al-Sulaiti et al., 2019; Chaudhary and Mudgal, 2020a; Devi and Rajendran, 2023; Jeong et al., 2021). Indian fermented foods have been shown to hypoglycemic effects by various mechanisms such as inhibition of enzymes involved in carbohydrate metabolism such as *α*-amylase and α-glucosidase (Chaudhary and Mudgal, 2020a; Chandana Kumari et al., 2022; Sreepathi et al., 2023), increasing insulin sensitivity (Hor et al., 2022), decreasing hyperinsulinemia (Hor et al., 2022; Yadav et al., 2006), antioxidant activity (Modi et al., 2023; Yadav et al., 2007), and anti-adipogenic activity (de Lopes et al., 2019; Hor et al., 2022). As shown in Table 2, certain mechanisms of action such as inhibition of α-amylase and α-glucosidase, antioxidant activity and anti-adipogenic activity are putatively attributed to probiotic-derived metabolites while the other mechanisms are assumed to be due to the probiotic strains. The antidiabetic activity of Indian fermented food with evidence from literature is elaborated in the following sections in accordance with their mechanism of action. Table 4 provides a summary of the key findings of studies on Indian fermented food with antidiabetic potential. Supplementary Table S2 elaborates on the methodology adopted in each of the studies in detail and reiterates the key findings of these studies.

**Table 4:** Summary of key highlights of studies on antidiabetic effects of various Indian fermented foods and their probiotic strains.

S. No	Indian fermented food	Probiotic strain	Methodology of the study	Antidiabetic activity reported	Reference
1	Fermented rice	*Bifidobacterium sp., Lactobacillus sp.*	*In Vivo* study using rats fed with normal diet and High Fat diet to induce diabetes.	Reduced hyperinsulinemia, improved insulin sensitivity due to expression of GLUT4 receptors in liver	Hor et al. (2022)
2	Milk-millet composite probiotic fermented product (finger millet)	*Lactobacillus helveticus* MTCC 5463	*In vitro* study using probiotic strain	Inhibition of enzymes (α-amylase and α-glucosidase)	Chaudhary and Mudgal (2020a)
3	Millet-legume-based Indian traditional fermented product	*Lactobacillus plantarum, Lactiplantibacillus plantarum, Enterococcus faecalis, Saccharomyces cerevisiae*	*In vivo* study using normal rats and streptozotocin-induced diabetic rats fed with a basal diet and experimental diet	Anti-hyperglycemic and Anti-nephropathic activity	Devi and Rajendran (2023)
4	Sprouted and fermented quinoa	Not mentioned	*In vivo* study using rats fed with control diet and high glycemic index diet to induce diabetes.	Decrease in glycemic index of diet and blood glucose levels	de Lopes et al. (2019)
5	Fermented amla beverage (Gooseberry)	*Pediococcus lolii, Lactobacillus plantarum, Pediococcus acidilactici, Pediococcus pennaceous*	*In vivo* study using normal rats and streptozotocin-induced diabetic rats.	Hypoglycemic effects and anti-oxidative activity	Modi et al. (2023)
6	Finger millet -enriched probiotic fermented milk	*Lactobacillus helveticus* MTCC 5463	*In vivo* study using non-diabetic rats and diabetic rats	Inhibition of enzymes (α-amylase and α-glucosidase)	Chaudhary and Mudgal (2020b)
7	Dahi (fermented milk product)	*Lactobacillus acidophilus, Lactobacillus lactis, Lactobacillus casei*	*In vivo* study using male albino Wistar rats fed with standard diet and high fructose diet to induce diabetes	Low GI index, delayed glucose intolerance and anti-oxidative activity	Yadav et al. (2007)
8	Fermented papaya	*Levilactobacillus brevis* RAMULAB52	*In vitro* study using probiotic strain	Inhibition of enzymes (α-amylase and α-glucosidase)	Sreepathi et al. (2023)
9	Skim Milk and Dahi (Yogurt)	*Lactococcus lactis s*sp. *lactis, L. lactis ssp. cremoris, L. lactis ssp. diacetylactis, Leuconostoc citrovorum*	*In vivo* study using rats fed with high fructose diet to model diabetic rats	Anti-hyperglycemic activity, reduced hyperinsulinemia	Yadav et al. (2006)
10	Dosa batter	*Limosilactobacillus fermentum* and *Lactisaseibacillus casei*	*In vitro* study using probiotic strain	Inhibition of enzymes (α-amylase and α-glucosidase) and radical scavenging activity	Chandana Kumari et al. (2022)
11	Fermented buffalo and camel Milk	*Limosilactobacillus fermentum* (KGL4), *Saccharomyces cerevisiae* (WBS2A)	*In vitro* study using probiotic strain	Inhibition of enzymes (α-amylase and α-glucosidase) and lipase	Khakhariya et al. (2023)
12	Fermented jalebi, medhu vada, and kallappam batters	*Lacticaseibacillus rhamnosus* RAMULAB13, *Lactiplantibacillus plantarum* RAMULAB14, *Lactiplantibacillus pentosus* RAMULAB15, *Lacticaseibacillus paracasei* RAMULAB16, *Lacticaseibacillus casei* RAMULAB17, *Lacticaseibacillus casei* RAMULAB20, *Lacticaseibacillus paracasei* RAMULAB21	*In vitro* study using probiotic strain	Inhibition of enzymes (α-amylase and α-glucosidase) and radical scavenging activity	Huligere et al. (2023)

Several studies have reported the hypoglycemic potential of various Indian fermented food. In an *in vivo* study where rats on high fructose diet were fed with fermented milk product called ‘dahi’ rich in probiotic bacteria *Lactobacillus acidophilus*, *Lactococcus lactis* and *Lactobacillus casei,* a reduced fasting blood glucose and glycated hemoglobin (HbA1c) levels were observed, suggesting its potential to prevent or delay the onset of hyperglycemia (Yadav et al., 2007). In another study, rats fed with ‘dahi’ rich in probiotic strains, *Lactococcus lactis s*sp. *lactis, L. lactis s*sp. *cremoris, L. lactis s*sp. *diacetylactis, and Leuconostoc citrovorum*, lowered blood glucose levels and HbA1c levels by 10% when compared to the control animals (Yadav et al., 2006). In an *in-vivo* study using diabetic rats, a 4-week feeding routine of Indian fermented food containing ‘whole finger millet with seed coat matter’ significantly reduced blood glucose levels by 40% in diabetic rats. It also lowered HbA1c levels by 41%, indicating improved glycemic control (Devi and Rajendran, 2023). Another potential fermented food that showed hypoglycemic effect is ‘fermented amla beverage’ rich in probiotic strains- *Pediococcus lolii, Lactobacillus plantarum, Pediococcus acidilactici* and *Pediococcus pennaceous.* Diabetic experimental rats that were given ‘fermented amla beverage’ showed a significant reduction in fasting blood glucose levels compared to the diabetic standard group, which received the hypoglycemic drug glibenclamide (Modi et al., 2023). In a similar study, ‘finger millet enriched probiotic fermented milk’ containing the probiotic strain *Lactobacillus helveticus* MTCC 5463 resulted in reduced blood glucose levels in diabetic rats when compared to the control group (Chaudhary and Mudgal, 2020b). Various other studies demonstrate the hypoglycemic effects of Indian fermented food such as ‘milk-millet composite’ and ‘fermented quinoa’ that significantly reduced plasma glucose, glycemic index, and HbA1c levels (Chaudhary and Mudgal, 2020a; de Lopes et al., 2019). These findings collectively highlight the potential of Indian fermented foods in managing blood glucose levels.

Studies have also revealed the hypoglycemic mechanisms of these fermented foods. The prominent mechanism has been found to be the presence of bioactive metabolites in the fermented foods that strongly inhibit carbohydrate digesting enzymes such as *α*-amylase and α-glucosidase leading to lower blood glucose levels. Another mechanism of hypoglycemic activity is the activation of GLUT4 transporters on muscle cells to absorb glucose from the blood, leading to lower blood glucose levels. A study evaluating an Indian ‘milk-millet composite fermented product’ showed significantly higher α-glucosidase inhibition and antioxidant activity, due to the polyphenols and dietary fiber in finger millet. Indian finger millet is rich in polyphenols such as hydroxybenzoic acids, hydroxycinnamic acids and flavonoids (Chethan and Malleshi, 2007). These components inhibit *α*-amylase and α-glucosidase lowering the enzymatic hydrolysis of complex carbohydrates and forming unabsorbable complexes, reducing carbohydrate absorption and controlling blood glucose levels (Chaudhary and Mudgal, 2020a). Another study identified probiotic bacteria from fermented ‘dosa batter’ that inhibit α-glucosidase and α-amylase. Out of 40 isolates, *Limosilactobacillus fermentum* RAMULAB11 exhibited substantial α-glucosidase and α-amylase inhibition. The inhibition of α-glucosidase ranged from 7.50 to 65.01%, while the inhibition of α-amylase ranged from 20.21 to 56.91% (Chandana Kumari et al., 2022). Inhibition of carbohydrate hydrolysis enzymes was also demonstrated through *in vitro* experiments using the probiotic strain, *Levilactobacillus brevis* RAMULAB52, isolated from ‘fermented papaya’. Cell free supernatant of the probiotic strain demonstrated 86.97 and 75.87% inhibition of the activities of α-glucosidase and α-amylase, respectively (Sreepathi et al., 2023). In another study, Khakhariya et al., 2023, showed that probiotic strains isolated from ‘fermented camel milk’ displayed high alpha-glucosidase and alpha-amylase inhibitory activities of 77.96% ± 2.61 and 70.86% ± 1.02, respectively. This was attributed to production of bioactive peptides (10–75 KDa) by the probiotic strains (Khakhariya et al., 2023). Several probiotic LAB isolated from ‘fermented jalebi batter’, ‘medhu vada batter’, and ‘kallappam batter’ exhibited α-glucosidase inhibition ranging from 15.08 to 59.55% and α-amylase inhibition varying between 18.79 and 63.42%. The strain *Lactiplantibacillus pentosus* RAMULAB15 demonstrated the highest inhibition rate for both α-glucosidase and α-amylase, with values of 59.55 and 63.42%, respectively (Huligere et al., 2023). In an independent study, ‘Indian fermented milk enriched with finger millet’ demonstrated significant antidiabetic effects. The product demonstrated inhibition of α-amylase and α-glucosidase, with inhibition percentages of 51.89 and 52.37%, respectively, indicating its potential in managing diabetes. *Lactobacillus helveticus* MTCC 5463 present in the fermented milk can enhance the release of bioactive peptides and amino acids. These compounds could directly affect pancreatic *β*-cells to stimulate insulin release and improve glucose uptake by cells through the activation of GLUT4 transporters (Chaudhary and Mudgal, 2020b). However, the metabolites responsible for the inhibition of α-glucosidase and α-amylase in most of the above-described studies (Chandana Kumari et al., 2022; Huligere et al., 2023; Sreepathi et al., 2023) have not been identified and this should be a focus of future research.

Hyperglycemia leads to increased reactive oxygen species which can cause lipid peroxidation, protein oxidation, and formation of advanced glycation end products. These effects eventually lead to insulin resistance, *β*-cell dysfunction and vascular complications such as retinopathy, nephropathy and neuropathy. Hence, several Indian fermented foods have been explored for their antioxidant activity in the context of treating diabetes. Yadav et al., 2007, conducted an *in-vivo* study where rats were fed with an Indian fermented milk formulation named ‘dahi’. The study revealed that dahi-supplemented diet alongside high fructose showed antioxidative effects in the liver and pancreatic tissues of diabetic animals by inhibiting thiobarbituric acid-reactive substances and the maintenance of reduced glutathione levels, indicating potential antidiabetic properties of ‘dahi’ (Yadav et al., 2007). In another study, the strain *Limosilactobacillus fermentum* (KGL4) from ‘fermented buffalo and camel milk’ exhibited significant antioxidative effects (Khakhariya et al., 2023). In a study using ‘finger millet’-enriched probiotic fermented milk’ containing *Lactobacillus* and streptococcal bacteria, significant antioxidant activity was observed due to the polyphenols present in the fermented food, including phenolic acids and flavonoids, which act as potent free radical scavengers (Chaudhary and Mudgal, 2020a). In another study, probiotic LAB (*Levilactobacillus brevis* RAMULAB52) isolated from ‘fermented papaya’ demonstrated notable antioxidative effects, by its ability to scavenge DPPH and ABTS radicals. *In vitro* and in silico analyses highlighted hydroxycitric acid as a key organic acid contributing to both its antioxidative and antidiabetic effects (Sreepathi et al., 2023). *In vitro* study using probiotic strains isolated from ‘dosa batter’, *Limosilactobacillus fermentum* and *Lactisaseibacillus casei*, displayed free radical scavenging activity in the range of 20.77–89.75%. The strain *Lactiplantibacillus pentosus* RAMULAB15 isolated from ‘fermented jalebi, medhu vada, and kallappam batters’ displayed 76.65% radical scavenging activity during *in vitro* studies (Huligere et al., 2023). Hence, with further research, the antioxidant potential of the above-mentioned Indian fermented food can be exploited as an adjunct to the conventional treatment methods for diabetes.

There is increasing evidence of fermented food conferring anti-inflammatory effects in diabetes by inhibiting pro-inflammatory cytokines. A study using *Lactobacillus kefiri* K and *Lactobacillus kefiranofaciens* M from ‘milk kefir’, Caucasus-region traditional diet, has shown to slow down the development of type 1 diabetes by hindering pro-inflammatory and inflammatory cytokines while boosting IL-10 production. IL-10 reduces pro-inflammatory cytokines like Th1 cytokines (IL-1*β*, IL-2, IL-6) and TNF-*α*, preventing the destruction of β cells (Wei et al., 2015). On similar lines, an Indian study using male C57BL/6 J mice treated with *Lactobacillus* species (MTCC 5690 and MTCC 5689) exhibited a substantial decrease in the proinflammatory gene expression patterns induced by a high-fat diet (Balakumar et al., 2018). Probiotic strains such as *Limosilactobacillus fermentum* (KGL4) and *Saccharomyces cerevisiae* (WBS2A) present in fermented ‘Indian buffalo or camel milk’ also have shown anti-inflammatory effects (Khakhariya et al., 2023). However, the mechanism by which the anti-inflammatory effect is conferred by the probiotic strain is not understood.

Obesity and excessive fat accumulation are strongly associated with a higher incidence of diabetes mellitus. In obesity, fats and lipids that are usually stored in adipocytes are deposited in the kidneys, liver, pancreas, muscles, and the bloodstream. Lipid accumulation within pancreatic β-cells impairs insulin secretion, whereas lipid deposition in skeletal muscle tissue diminishes insulin sensitivity by disrupting the function of glucose transporters. Hence, preventing accumulation of excess fat and lipid in the body through anti-adipogenesis or inhibition of lipase in the gut that can lower lipid absorption in the body can help the treatment success of diabetes. On these lines, ‘Indian fermented rice’ prepared using the root dust of *Asparagus racemosus*, rich in *Lactobacillus* sp., showed significant anti-obesity effects in mice fed with a high-fat diet. Supplementation of ‘Indian fermented rice’ reduced body weight by 26%, improved lipid profiles, and mitigated fatty liver. Also, the mice fed with the ‘Indian fermented rice’ exhibited a significant improvement of 77.89% in adiponectin levels when compared to the control group. Since adiponectin hormone can enhance fatty acid oxidation, it can lower lipid levels and improve insulin sensitivity (Hor et al., 2022). Another Indian study showed that the fermentation process enhances quinoa’s ability to mitigate adipogenesis in Wistar rats. Diets incorporating fermented quinoa significantly reduced lipid levels, blood glucose, and the accumulation of epididymal adipose tissue. These anti-adipogenic effects are attributed to the increased dietary fiber content and the chemical transformations occurring during quinoa fermentation (de Lopes et al., 2019). In an independent study, feeding rats on high fructose diet with ‘dahi’ reduced serum lipid levels along with glucose levels (Yadav et al., 2007). Another study showed that probiotic strains in ‘fermented camel milk’ displayed 85.37% ± 2.15 of lipase inhibitory activity. Inhibition of lipase in the gut is beneficial as it can lower lipid absorption from diet into the body. This was attributed to production of bioactive peptides (10–75 KDa) by the probiotic strains (Khakhariya et al., 2023). Hence, these studies demonstrate the ability of Indian fermented foods to confer anti-adipogenic and lipase inhibitory activity in the context of diabetes management. The signaling cascade involved in the anti-adipogenic activity of probiotics organism or specific metabolites produced by the probiotic strains should be understood through further research.

In a similar study from the same group, ‘dahi’ consumption significantly reduced glucose intolerance and hyperinsulinemia in rats with high fructose diet-induced type 2 diabetes (Yadav et al., 2006). ‘Dahi’ was found to be rich in probiotic strains such as *Lactococcus lactis ssp. lactis, Lactococcus lactis ssp. cremoris, Lactococcus lactis ssp. diacetylactis, and Leuconostoc citrovorum.* Plasma insulin levels were notably reduced by 48% in animals fed with dahi, compared to the control group. On these lines, the consumption of ‘Indian fermented rice’ prepared using the root dust of *Asparagus racemosus*, has shown promising effects on insulin sensitivity. ‘Indian fermented rice’ prepared using the root dust of *Asparagus racemosus* as a starter component significantly improved glucose metabolism and glucose tolerance in obese mice. This improvement was a result of enhanced levels of glucose metabolism-related hormones and increased insulin sensitivity, due to higher expression of GLUT4 receptors in liver cells. It also showed reduction in body weight, BMI, and fat accumulation (Hor et al., 2022).

Diabetes mellitus leads to other health complications such as nephropathy, retinopathy, neuropathy and liver damage. A few studies on the antidiabetic potential of Indian fermented food have shown anti-nephropathic and hepato-protective effects conferred by these foods. Histopathological studies of diabetic rats fed with ‘fermented amla beverage’ containing *Pediococcus lolii, Lactobacillus plantarum, Pediococcus acidilactici* and *Pediococcus pennaceous* strains showed reduced liver damage. The hepatocytes of these rats showed no fat vesicles or inflammation, and the cellular structure remained intact (Modi et al., 2023). A similar study on the effect of ‘finger millet enriched fermented milk’ rich in *Lactobacillus helveticus* MTCC 5463 on diabetic rats revealed that the fermented food supplementation partially improved the inflammation and changes in liver structure observed in diabetic rats during histopathological analysis (Chaudhary and Mudgal, 2020b). In an independent study where ‘millet-legume based fermented product’ rich in *Lactobacillus plantarum, Lactiplantibacillus plantarum, Enterococcus faecalis* and *Saccharomyces cerevisiae* were fed to diabetic rats, histopathological analysis revealed a normal glomerulus with minimal thickening in the mesangial cells, indicating the anti-nephropathic effect of the fermented food (Devi and Rajendran, 2023).Probiotic strains from various fermented food tested for their antidiabetic effects also confer additional benefits such as antibacterial activity against food-borne pathogens and intestinal colonization ability which can reduce possible dysbiosis in the gut. *Lacticaseibacillus rhamnosus* RAMULAB13, *Lactiplantibacillus plantarum* RAMULAB14 strains isolated from ‘idli’, a rice-based fermented food from southern part of India, exhibited auto and coaggregation abilities, enhancing their colonization and interaction in the gut microbiota (Huligere et al., 2023). They also demonstrated hydrophobicity, facilitating adhesion to intestinal mucosa for improved efficacy. Moreover, these isolates display significant antibacterial features, contributing to the maintenance of a balanced gut microbiome and potentially aiding in diabetes management (Huligere et al., 2023). A similar study employing probiotic strains, *Limosilactobacillus fermentum* and *Lacticaseibacillus casei,* isolated from ‘dosa batter’ demonstrated promising antimicrobial activity against *Micrococcus luteus* and *Pseudomonas aeruginosa* (Chandana Kumari et al., 2022). In another study, the cell-free supernatant of *Levilactobacillus brevis* RAMULAB52, isolated from ‘fermented papaya’ exhibited anti-bacterial activity. The strain also showed adhesion to different cells such as buccal epithelial cells, HT-29 cells and chicken crop epithelial cells which indicates its ability of intestinal colonization.

Hence, anti-diabetic fermented foods and the associated probiotics provide a multitude of beneficial effects such as hypoglycemic activity, anti-dyslipidemia, anti- adipogenesis activity, antioxidant activity, anti-inflammatory activity, increased insulin sensitivity and reduced nephropathy and liver damage, which can help treat diabetes in a holistic manner. Future research must aim to validate these findings in human through clinical trials. As iterated at various instances in the above discussion, future work should also focus on understanding the mechanism of action of probiotics or metabolites produced by probiotics present in fermented foods.

## 5 Anticholesterolemic activity of Indian fermented foods

Cholesterol is an essential lipid involved in various physiological functions, but hypercholesterolemia, characterized by elevated levels of total cholesterol, low-density lipoprotein cholesterol (LDL-C), and high-density lipoprotein cholesterol (HDL-C) is a major risk factor for coronary artery diseases (CAD) and stroke (Pirillo et al., 2021). Hypercholesterolemia is the eighth leading cause of death in 2019. In 2019, 3.78 million deaths due to CAD and 0.61 million deaths due to stroke were attributed to hypercholesterolemia (Pirillo et al., 2021).

In the Indian context, several fermented Indian foods have been demonstrated to confer anticholesterolemic effect (Satish Kumar et al., 2013). Probiotic strains from Indian fermented food have shown to influence cholesterol metabolism by deconjugating bile salts (Anandharaj et al., 2015; Ray et al., 2018), by adsorbing or assimilating cholesterol (Anandharaj et al., 2015; Bhat et al., 2019) or by conferring hepatoprotective effects via improving the activity of ROS scavenging enzymes in the liver (Yadav et al., 2019). All these hypocholesterolemic activities are attributed to the probiotic rather than metabolites produced by the probiotic organism as shown in Table 2. The following paragraphs elaborate the mechanism of action and hypocholesterolemic activity of various Indian fermented food evaluated using *in vitro* and *in vivo* studies. Table 5 provides a summary of the major highlights of studies on anticholesterolemic activity of Indian fermented food. Supplementary Table S3 elaborates the methodology adopted in each of the studies in detail and reiterates the key findings of these studies.

**Table 5:** Summary of key highlights of studies on anticholesterolemic effects of various Indian fermented foods and their probiotic strains.

S. No	Indian fermented food	Probiotic strain	Methodology of study	Anticholesterolemic activity reported	References
1	Fermented rice	*Bifidobacterium* sp. MKK4	*In vivo* study using rats fed High Fat diet.	Cholesterol-lowering activity, BSH activity, restoring liver function	Ray et al. (2018)
2	Fermented koozh (rice-based food) and gherkin (fermented cucumber)	*Weissella koreensis* FKI21, *Lactobacillus crispatus* GI9	*In vitro* study using probiotic strains	Cholesterol-lowering activity, BSH activity, cholesterol assimilation from growth media	Anandharaj et al. (2015)
3	Kalarei (Fermented milk product)	*Enterococcus faecium* K2	*In vitro* study using probiotic strains	Cholesterol-lowering activity, BSH activity, cholesterol adsorption to cell membrane	Bhat et al. (2019)
4	Yogurt	*Enterococcus faecium* Chloe1, *Enterococcus faecium* EF, *Lactobacillus pentosus* 7MP	*In vitro* study using probiotic strains	Cholesterol-lowering activity, BSH activity	Walhe et al. (2021)
5	Fermented Indian food	*Pediococcus pentosaceus* G11, *Pediococcus pentosaceus* AP3	*In vitro* study using probiotic strains	Cholesterol-lowering activity, BSH activity	Rajan et al. (2021)
6	Yogurt	*Bifidobacterium bifidum* 2715	*In vivo* study using rats fed with basal diet and cholesterol diet	Serum cholesterol and LDL lowering activity	Athrayilkkalathil and Prasad (1997)
7	Probiotic dahi (Fermented milk product)	*Lactobacillus fermentum* strains, i.e., PH5 (handva batter isolate) and PD2 (dosa batter isolate).	*In vivo* study using rats fed with normal diet and hyperlipidemic diet	Total-cholesterol and LDL lowering activity, reduced hepatocyte steatosis	Thakkar et al. (2020)
8	Fermented cereal-pulse food mixture	*Lactobacillus casei* (NCDC-19), *Saccharomyces boulardii*	*In vivo* study using rats fed with unfermented food with 1% cholesterol and rats fed with fermented food mix with 1% cholesterol	Serum and liver cholesterol lowering activity, serum LDL lowering activity	Sindhu and Khetarpaul (2003)
9	Fermented milk	*Lactobacillus rhamnosus* 5,957, *Lactobacillus rhamnosus* 5,897	*In vivo* study using rats fed with standard diet and high cholesterol diet	Serum triglycerides cholesterol and LDL-lowering activity, improved catalase and superoxide dismutase activity in liver	Yadav et al. (2019)

Several *in-vivo* studies have demonstrated the cholesterol-lowering ability of Indian fermented food. An *in vivo* study where ‘Indian fermented rice’ containing the probiotic strain *Bifidobacterium* sp. MKK4 was fed to high fat diet induced obese mice models, 53% reduction in cholesterol levels, reduced body weight, improved lipid profiles, and decreased inflammation was observed. Anti-inflammatory and antioxidative effects were significant, highlighting their potential in managing obesity-related metabolic disorders (Ray et al., 2018). In a similar study, when Indian fermented milk product, ‘dahi’, with the probiotic strain *Lactobacillus fermentum* PH5 was administered to rats fed with hyperlipidaemic diet, examined for serum cholesterol level, LDL and HDL levels showed reduced total cholesterol level by 67% and LDL cholesterol level by 63%. The study also demonstrates anti-inflammatory and antioxidant properties (Thakkar et al., 2020). When ‘Indian fermented cereal-pulse mixture’ rich in the probiotic strains *Lactobacillus casei* (NCDC-19) and *Saccharomyces boulardii* was fed to mice, showed hypocholesterolemic effect with 19% reduction in total serum cholesterol level, 11% increase in HDL level and 37% decline in LDL level in the experimental group (Sindhu and Khetarpaul, 2003). Another study showed that ‘dahi’ fermented with *Lactobacillus rhamnosus* (LR 5957 and LR 5897) strains shows lowered cholesterol and LDL levels when fed to hypercholesterolemic rats (Yadav et al., 2019).

Several studies have demonstrated bile salt hydrolase (BSH) activity of the probiotics present in Indian fermented food which is considered a dominant mechanism of hypocholesterolemic effects of these fermented foods (Anandharaj et al., 2015; Athrayilkkalathil and Prasad, 1997; Ray et al., 2018; Rajan et al., 2021; Walhe et al., 2021). BSH deconjugates bile salts to free bile acids that leads to its lower solubility and easy elimination from the intestine. Since cholesterol is a major precursor in the bile acid synthesis, elimination of bile acids can lead to increased bile acid synthesis in the body thereby reducing cholesterol levels. *Bifidobacterium* sp. MKK4 isolated from ‘fermented rice’ was found to possess BHS gene and also showed BSH activity *in vitro*. The administration of the fermented rice to rats resulted in 53–56% reduction in serum cholesterol levels (Ray et al., 2018). An *in vitro* study using probiotic strains- *Lactobacillus crispatus* GI9 and *Weissella koreensis* FKI21 isolated from Indian fermented rice-based food, ‘koozh’ and ‘gherkins’ (fermented cucumber) were tested for their ability to reduce cholesterol levels, survive gastrointestinal conditions, and exhibit bile salt hydrolase activity. The study showed that the probiotic strains displayed high cholesterol reduction and were able to deconjugate bile salts such as sodium glycocholate and sodium taurocholate (Anandharaj et al., 2015). In another study, *Enterococcus faecium* K2 strain isolated from Indian fermented milk product, ‘kalarei’, showed strong bile salt deconjugation potential, strong antioxidant activity and high *in vitro* cholesterol lowering ability (82.32%) (Bhat et al., 2019). *Lactobacillus pentosus* (7MP) strain isolated from ‘yoghurt’ was positive for BSH activity and demonstrated a cholesterol reduction activity of 47.32% (Walhe et al., 2021). The strain G11 and AP3 of *Pediococcus pentosaceus* isolated from fermented Indian food shows BSH activity and reduction in cholesterol level of 40 to 50% (Rajan et al., 2021).

Studies have also revealed that the cholesterol-lowering ability of probiotics can also be attributed to their ability to assimilate cholesterol into the cell or adsorb cholesterol on the cell membrane (Anandharaj et al., 2015; Bhat et al., 2019). An *in vitro* study using *Weissella koreensis* FKI21 and *Lactobacillus crispatus* G19 isolated from ‘koozh’ and ‘gherkin’ demonstrated a notable decrease in cholesterol levels when the growth media of the probiotic strains was enriched with 50 mg/mL of cholesterol, thus showing the ability of the probiotic strains to assimilate cholesterol into their cells (Anandharaj et al., 2015). In another *in vitro* study, *Enterococcus faecium* K2 isolated from ‘kalarei’ exhibited a high capacity to attach cholesterol to its cellular membrane and showed cholesterol reduction capability of 82.32% (Bhat et al., 2019).

Certain anticholesterolemic Indian fermented food have also found to display antioxidant properties. Since reactive oxygens species (ROS) can oxidize LDL-cholesterol and oxidized LDL-cholesterol can promote atherosclerosis, antioxidant activity of fermented food can prevent the occurrence of atherosclerosis which is a common outcome of prolonged hypercholesterolemia (Yadav et al., 2019). Administration of ‘fermented milk’ rich in *Lactobacillus rhamnosus* 5,957 or *Lactobacillus rhamnosus* 5,897 also led to increase in catalase and superoxide dismutase activity in liver tissue. Catalase activity was significantly enhanced by the probiotic strains LR 5957 and LR 5897, indicating strong antioxidant effects. However, only LR 5957 exhibited a significant 1.3-fold increase in superoxide dismutase activity compared to the HCD group. There was no statistically significant increase in glutathione peroxidase activity in the probiotic fermented milk (Yadav et al., 2019).

In the context of cholesterol management, a few studies on Indian fermented food have evaluated the liver-protective effects of these foods. In an *in vivo* study where ‘dahi’ rich in *Lactobacillus fermentum* strains PH5 and PD2 was fed to hyperlipidemic rats, histopathological study of the liver revealed reduced hepatocyte steatosis (Thakkar et al., 2020). In another *in vivo* study, when ‘fermented rice’ rich in the probiotic strain, *Bifidobacterium* sp. MKK4, was fed to rats on high-fat diet, the levels of SGPT (serum glutamic-pyruvic transaminase) and SGOT (serum glutamic- oxaloacetic transaminase) were restored to normal, indicating the liver-protective potential of the fermented food.

Hence, Indian fermented food provides an array of anticholesterolemic effects and further research involving more animal studies and human trials is required to translate these finding to clinical settings. Research must also focus on deciphering the mechanism of action of probiotic containing fermented food. While Indian fermented foods such as ‘fermented rice’, ‘kalerei’ display hypocholesterolemic effects through BSH activity and cholesterol assimilation (Anandharaj et al., 2015; Bhat et al., 2019), others like ‘fermented milk’ are found to reduce cholesterol levels by improving activity ROS scavenging enzymes in liver tissue (Yadav et al., 2019). However, the mode of action of Indian fermented foods such as ‘yogurt’ and ‘fermented cereal-pulse food mixture’ still remain poorly understood (Athrayilkkalathil and Prasad, 1997; Sindhu and Khetarpaul, 2003). Research in this direction could facilitate the utilization of these fermented foods in the development of effective prophylactic and therapeutic applications.

## 6 Antibacterial activity of Indian fermented foods

In 2001, three bacterial infections, viz., lower respiratory tract infections (3.4 million deaths), diarrheal diseases (1.8 million deaths) and tuberculosis (1.6 million deaths) were ranked among the top five infections that caused the highest number of deaths in low- and middle-income countries (Michaud, 2009). Pathogenic bacteria such as *Klebsiella pneumoniae*, *Streptococcus pneumoniae*, *Staphylococcus aureus*, *Pseudomonas aeruginosa* cause lower respiratory tract infections while diarrheal diseases are caused by the infection of the intestinal tract by food and water-borne bacteria such as *Escherichia coli*, *Salmonella* species, *Shigella* species, etc. Tuberculosis is caused by the bacteria *Mycobacterium tuberculosis* which resulted in 1.23 million deaths worldwide in 2024 (World Health Organization, 2026b).

Several studies have reported the antibacterial and antiviral activity of Indian fermented food. Antibacterial activity of probiotic strains is attributed to the capability of these strains to produce different kinds of antibacterial compounds that include bacteriocins, bacteriocin-like inhibitory substances (BLIS), low molecular weight antibacterial compounds and other uncharacterized compounds. However, the mechanisms underlying the antibacterial activity of these compounds remain poorly understood, with the exception of certain bacteriocins that have been reported to induce cell lysis through pore formation in the bacterial cell wall as shown in Table 2. The following paragraphs elaborate on the evidence and the understanding of mechanism of action of these fermented foods. Table 6 enlists some of the Indian fermented foods displaying antimicrobial activity with details of the isolated probiotic strain, methodology employed and key finding of each study. Supplementary Table S4 elaborates the methodology adopted in each of the studies in detail and reiterates the key findings of these studies.

**Table 6:** Summary of key highlights of studies on antimicrobial activity of various Indian fermented foods and their probiotic strains.

S. No	Indian fermented food	Probiotic strain	Methodology of the study	Antimicrobial property reported	References
1	Tungtap (fermented fish) and Tungrymbai (fermented soybean)	*Lactobacillus plantarum, Lactobacillus brevis*, *Lactobacillus casei*, *Lactobacillus fermentum*, *Pediococcus pentosaceus*, *Enterococcus* sp.	*In vitro* study using the cell-free supernatants (CFS) derived from the LAB strains	Production of bacteriocins	Biswas et al. (2017)
2	Dosa batter (fermented cereal-pulse based food)	*Lactobacillus plantarum* LD4	*In-vitro* study using the CFS obtained from an overnight culture of *Lactobacillus plantarum* strain LD4.	Production of bacteriocins	Kumar et al. (2016)
3	Uttappam batter, dosa batter (fermented cereal-pulse based food)	*Lactobacillus rhamnosus, Lactobacillus plantarum, Lactobacillus brevis*	*In vitro* study using CFS of the isolated cultures.	Production of anti-bacterial low molecular weight compound (2-hydroxyl indole-3-propanamide)	Jeevaratnam et al. (2015)
4	Hentak (traditional fermented fish product)	*Lactobacillus pentosus*	*In vitro* study using CFNS of the isolated strain.	Production of uncharacterized substances with antibacterial properties	Aarti et al. (2016)
5	Kinema (fermented soybean food), Dahi (fermented milk product)	*Lactobacillus plantarum* DHCU70, *Lactobacillus plantarum* DKP1	*In vitro* study using the CFS of the isolated strains.	Production of bacteriocins inhibiting cell wall synthesis	Goel et al. (2020)
6	Shidal (salted fermented fish food)	*Lactobacillus plantarum* LA21	*In vitro* study using cell-free extract of *L. plantarum* LA21	Production of bacteriocins	Leslie et al. (2021)

Bacteriocin is a common antimicrobial peptide produced by various probiotic strains. Plantaricin, a bacteriocin produced by *Lactobacillus plantarum* DHCU70 isolated from ‘dahi’ (Indian fermented milk product), and *Lactobacillus plantarum* DKP1 isolated from kinema (a soya-bean based fermented food from North-East India), have shown to inhibit pathogenic bacteria, *Kocuria rhizophila* ATCC 9341. The mode of inhibition of plantaricin was found to be cell wall inhibition from reporter assays (Goel et al., 2020). Bacteriocins are commonly known for their inhibition toward gram-positive bacteria, and they show relatively less control toward gram-negative bacteria (Darbandi et al., 2022). However, a bacteriocin, produced by *Lactococcus lactis subsp. lactis* strain 63, isolated from Indian fermented dairy products (such ‘cream’, ‘dahi’, ‘yogurt’, ‘cultured milk’, and ‘flavoured milks’), has shown potential control against gram-negative bacteria such as *E. coli, Yersinia, Citrobacter, Proteus, Enterobacter, Klebsiella and Serratia strains* (Goyal et al., 2018). A study revealed that bacteriocin secreted by *L. plantarum* LD4 screened from ‘dosa batter’ (a rice-based fermented food from South India), acts as a broad-spectrum bactericidal substance with pore formation as a mode of inhibition (Kumar et al., 2016). In the light of existing nosocomial infection, which adds on to the fatal multidrug resistance (MDR) (van Duin and Paterson, 2016), a bacteriocin, isolated from Indian fermented food – ‘Tungtap’, has been studied for its inhibitory effect on *β*-Lactamase producing nosocomial bacteria individually and synergically with antibiotics such as cefotaxime, imipenem, tigecycline and polymyxin B. The minimum inhibitory concentration was found to be in the range of 6.66 to 26.66 mg/mL, making it a potential future candidate against MDR infections (Biswas et al., 2017).

Antibacterial peptides that are not fully characterized are categorized as bacteriocin-like inhibitory substances (BLIS). A bacteriocin-like molecule, produced by *L. plantarum* LA21 strain isolated from ‘shidal’, a fish-based fermented food from North-East part of India, was found to inhibit the growth of various foodborne pathogenic bacteria such as *Listeria monocytogenes, Bacillus amyloliquefaciens,* and *Staphylococcus aureus,* which are already proven to have multidrug resistance. Further, the bacteriocin-like molecule was shown to display competent molecular interaction with bacterial surface proteins using molecular docking (Leslie et al., 2021).

Other than antibacterial peptides, metabolomic analysis of cell-free supernatants of cultures isolated from fermented food have been discovered to produce antibacterial compounds. 2-Hydroxyl Indole-3-Propanamide isolated from LAB strains of fermented ‘idli batter’ and ‘uttapam batter’ (a rice-based South Indian food), has shown broad spectrum antibacterial activity. With its low molecular weight (LMW), its possible application as an antibiotic need to be investigated (Jeevaratnam et al., 2015). There are also studies where the cell free supernatant of the cultures isolated from Indian fermented food contain uncharacterized substances with antibacterial properties. An antibacterial compound analyzed from ‘Hentak’ (a fermented fish product of North-East India), depicted elevated level of inhibition activity against enteric bacteria when cultured in MRS media with Tween 20 as a supplement (Aarti et al., 2016).

Hence, probiotics have promising capability against pathogenic diseases and can act as a potential alternative with extensive investigation and controlled fermentation. Although, majority of these studies have shown *in vitro* anti-microbial activity using cell-free supernatants of the probiotic strains isolated from fermented food, *in vivo* studies involving administration of fermented food to diseased animal models and tracing their recovery has not been done. Research along these lines can help in establishing fermented food as a treatment strategy for various infectious diseases.

Further, future research should focus on understanding the mechanism of action of these probiotic foods. Although the mechanism of action of bacteriocins present in certain Indian fermented foods like’ kinema’ (fermented soybean food), ‘dahi’ (fermented milk product) and ‘dosa batter’ has been understood as cell lysis resulting from pore formation in the cell membrane (Goel et al., 2020; Kumar et al., 2016), the mechanism of action of other antibacterial metabolites like BLIS, low molecular weight compounds and certain uncharacterized compounds found in Indian fermented foods like ‘tungtap’ (fermented fish), ‘uttappam batter’ (fermented cereal-pulse based food), ‘hentak’ (traditional fermented fish product), and ‘shidal’ (salted fermented fish food) is still not deciphered (Aarti et al., 2016; Jeevaratnam et al., 2015; Leslie et al., 2021). Future research should focus on elucidating the mechanisms of action of these metabolites, as this would facilitate the effective translation of their antibacterial properties into therapeutic interventions.

## 7 Commercialized probiotic strains vs. traditional fermented foods in India: a comparative

The Indian probiotic market predominantly features strains validated after stringent safety and efficacy trials. *Lactobacillus* species, such as *L. acidophilus*, *L. rhamnosus*, and *L. casei*, along with *Bifidobacterium* strains (*B. lactis*, *B. longum*), dominate commercial formulations (Kesavelu et al., 2020). These strains are selected for their ability to survive the transit across the gastrointestinal tract, adherence to intestinal mucosa, and conferral of other benefits like diarrhea prevention, lactose digestion, and immune modulation. For instance, Yakult’s proprietary strain *L. casei* Shirota is linked to improved gut barrier function (Liew et al., 2018; Macnaughtan et al., 2020).

**Figure 1:**
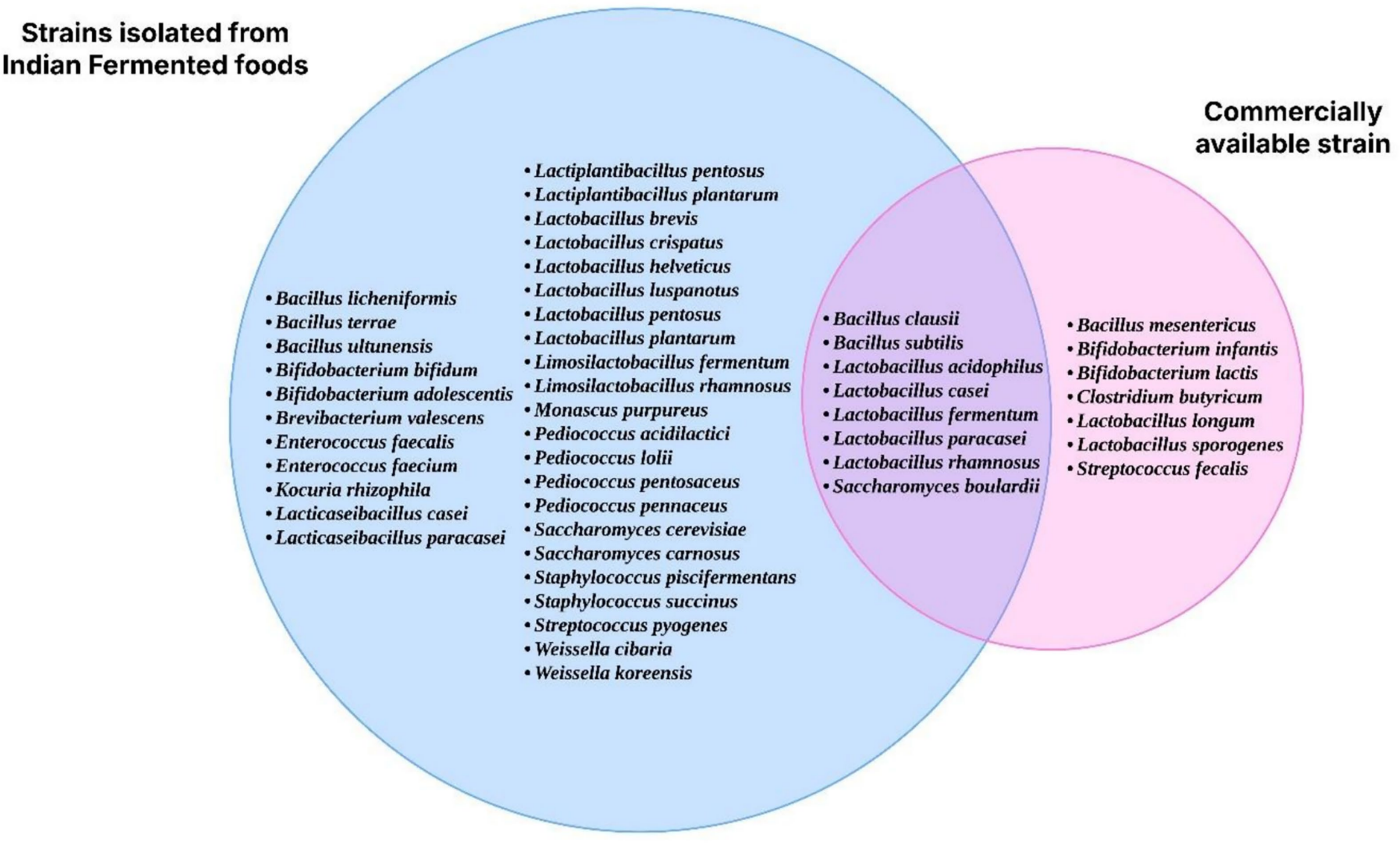
A comparison between traditionally isolated probiotic strains and commercially available ones.

Figure 1 shows the overlap between probiotic strains found in Indian fermented foods and those currently available in the market for consumption. It is seen from Figure 1 that a large proportion of the probiotics found in Indian fermented food have not been explored for commercialization.

While commercial probiotics prioritize strain-specific, dose-controlled formulations, traditional fermented foods offer greater microbial diversity, often acting in tandem with local dietary patterns and gut microbiome. For instance, *Lactobacillus plantarum* found in the traditional South Indian food ‘idli’ demonstrates anti-hyperglycemic effects but lacks clinical validation for the targeted health outcomes (Huligere et al., 2023). In contrast, commercial strains like *Bifidobacterium lactis* BB-12 are backed by trials confirming their role in activating immune cells (Merenstein et al., 2015).

A key limitation of traditional fermented foods is the absence of strain-specific characterization and regulatory oversight, despite their cultural acceptance. Commercial products often overlook regionally adaptable strains that may offer tailored benefits for Indian populations. Hence, the current study advocates for validating the health benefits conferred by indigenous strains from traditional foods through human clinical studies and to develop culturally resonant probiotics with better efficacy.

## 8 Traditional Indian fermented foods: a sustainable food processing strategy

Fermented food contributes heavily to India’s food system traditionally and industrially. Traditionally, rural and tribal communities have fermented local crops and milk for preservation and nutrition. In total, over 200 documented Indian fermented product have been reported in literature which reflects a heavy dependance of the Indian nutrition and diet on fermented food (Samantaray and Saha, 2026; Gabriele et al., 2026). From an industrial perspective, dairy fermentation is widespread: about 10% of India’s milk in organized sector and ~14% in informal sector is made into fermented food products such as curd, lassi, shrikhand, etc. (Government of India, Ministry of Food Processing Industries, 2021). Modern food companies now market traditional fermented snacks and drinks (e.g., packaged yogurt, fermented batter), aligning with global functional foods trends. These data show that fermentation is entrenched in Indian diets nationwide, and industry growth signals expanding use (Samantaray and Saha, 2026; Government of India, Ministry of Food Processing Industries, 2021).

Fermentation confers multiple sustainability advantages to the Indian food processing system making it nutritionally, environmentally, socially and economically sustainable. Nutritionally, fermentation enhances food value. It breaks down anti-nutritional factors (e.g., phytates in grains), making vitamins and minerals more absorbable. It increases the digestibility of food such as millets, legumes and milk (Suri and Ray, 2025; FAO, 2014). For example, lactic acid bacteria in dosa/curd hydrolyze lactose and proteins, alleviating intolerance and increasing bioavailability. Probiotic microbes in fermented food products improves gut health and can provide prophylactic and ameliorative effects against various diseases (Gul and Durante-Mangoni, 2024; Abouelela and Helmy, 2024). Hence, fermentation simultaneously preserves calories, improves digestibility, enhances nutrient bioavailability, and offers prophylactic and ameliorative effects against several diseases.

Microbial fermentation makes food processing environmentally sustainable in various ways. Microbial processing extends shelf life and reduces spoilage resulting in reduced food wastage (Suri and Ray, 2025; FAO, 2014). Food processing through fermentation is less energy intensive as microbial fermentation happens at ambient temperature and the fermented products could be stored without refrigeration (Suri and Ray, 2025; Gabriele et al., 2026). For instance, converting cereals and dairy into idli batter or yogurt preserves them at ambient temperature. Fermentation also enables resource circularity: food residues and surplus produce are upcycled rather than landfilled, cutting emission of green-house gases from decay or burning (Dhiman et al., 2025). Resource circulatory is also achieved in case of fermented foods such as beverages or dairy products where the by-products from the preparation of these fermented foods such as spent grains or whey are diverted as protein rich livestock feed supplies (FAO, 2014).

Indian fermented food system has also contributed to social and economic sustainability. Production of fermented food has helped preserve traditional knowledge and has provided employment to local communities that have the technical know-how of preparation of the fermented foods. Economically, the Indian fermented food and beverage sector represents a rapidly expanding market, valued at approximately USD 22.8 billion in 2024 and projected to reach USD 41.9 billion by 2033 (Samantaray and Saha, 2026). Furthermore, Indian fermented food exports account for nearly 2% of global processed food exports, highlighting their growing commercial significance in the international food market (Samantaray and Saha, 2026). Collectively, Indian fermented foods embody a sustainable food paradigm that supports sustainable nutrition, socio-economic well-being and environmental resilience.

## 9 Future outlook

Despite the growing body of research over the past two decades exploring the health-promoting potential of Indian fermented foods, several critical research thrust areas remain insufficiently addressed, thereby limiting their full translation into mainstream functional foods and adjunctive applications in clinical medicine. Firstly, comprehensive randomized human clinical trials to validate the health benefits of Indian fermented foods in humans are scarce in literature. Such human trials are a need of the hour to translate the health benefits of the Indian fermented foods into development of mainstream prophylactic or auxiliary treatment method for various diseases. Secondly, research must also focus on deciphering the mechanisms of action by which the probiotic containing fermented food is able to confer ameliorative effects in various disease contexts. Such understanding is crucial for establishing strain-specific functionality, optimizing dosage and formulation, and predicting clinical efficacy under different physiological and pathological conditions. Thirdly, although researchers have focussed on evaluating the anticancer, antidiabetic, antihypercholesterolemia and antimicrobial effects of Indian fermented food, there is a dearth of studies on the antipsychotic effects of Indian fermented food, which needs to be addressed through future research. Fourthly, there still exists a multitude of unexplored fermented food options from India and hence research in this direction will help identify more probiotic foods of Indian origin with therapeutic potential. Finally, bridging the gap between commercialized probiotics and India’s fermented foods through industry-academia collaborative research to validate traditional strains and integrating them into regulated products may enhance market relevance and public health impact.

Alongside advancements in research on Indian probiotics and fermented foods, concerted efforts from national regulatory and scientific agencies are essential to facilitate the translation of scientific findings into reliable and commercially viable products. To enhance the credibility of fermented foods and expand their acceptance in international markets, comprehensive safety, quality, and regulatory standards must be established and systematically implemented by national authorities (Samantaray and Saha, 2026; Suri and Ray, 2025; Bhatt and Joshi, 2025). Furthermore, the development of a centralized registry of traditional starter cultures could contribute significantly to the standardization and preservation of indigenous fermentation practices. Equally important is institutional support for small-scale artisanal producers through training in hygienic production practices, quality assurance, and regulatory certification pathways, thereby safeguarding traditional knowledge while ensuring product safety and marketability (Suri and Ray, 2025).

## 10 Conclusion

Over the past two decades, substantial research has focused on elucidating the health-promoting potential of Indian fermented foods containing probiotics. The therapeutic potential of these fermented foods against diseases such as cancer, diabetes, hypercholesterolemia, and microbial infections has been demonstrated through numerous *in vitro* investigations and *in vivo* studies employing animal models. Immunomodulation, production of antioxidants, improving expression of ROS scavenging enzymes in the host, production of bacteriocins and other antibacterial biomolecules, bile salt hydrolase activity, inhibition of *α*-amylase and α-glucosidase, metabolic transformation of carcinogens have been recognized as a few modes of action of the probiotics by which they confer these health benefits. Despite these promising findings, there remains a paucity of well-designed randomized controlled clinical trials to substantiate the reported health claims, which is critical for translating experimental evidence into evidence-based prophylactic and adjunctive therapeutic strategies for various diseases. Furthermore, stronger industry–academia collaborations aimed at the commercialization of traditional fermented foods, together with coordinated efforts from national regulatory agencies to establish comprehensive safety and quality guidelines, are essential for translating current scientific evidence into reliable, standardized, and safe commercial products.

Walking along the lines of Socrates himself “Let thy food be thy medicine and medicine be thy food” summarizes the essence of this article. However, there is a long way before fermented foods get incorporated into the treatment regimen against various diseases and with further research Indian fermented food can play a vital role in relieving mankind of these maladies.
