# Supplementary material for: Unlocking the health potential of fermented foods: insights from traditional Indian diets
Source: Front Microbiol. 2026 Jun 29;17:1860268. doi: 10.3389/fmicb.2026.1860268 (PMC13357518; doi:10.3389/fmicb.2026.1860268)
Supplement: Supplementary file 1 [file Data_Sheet_1.docx]

**Supplementary data**

**Table S1 Detailed methodology and results of studies on anticancer activity of Indian fermented foods**

| **S. No** | **INDIAN FERMENTED FOOD** | **PROBIOTIC STRAIN** | **METHODOLOGY OF THE STUDY** | **ANTICANCER ACTIVITY REPORTED** | **REFERENCE** |
| --- | --- | --- | --- | --- | --- |
| 1. | Tomato Pickle | *Pediococcus acidilactici* TMAB26 | In vitro study using the strains isolated from tomato pickle. The strains were tested for their cytotoxic effect, intestinal cell adhesion ability and anti-inflammatory effects in vitro using HT-29 cell lines. Anti-inflammatory effect was assessed by evaluating the expression levels of pro- and anti-inflammatory cytokines in the cell lines by qRT-PCR. | The isolated strain, *P. acidilactici* TMAB26, exhibited 94.91% cytotoxic activity against intestinal cancer cells and 92.63% against Caco-2 cancer cells.  Upregulation of IL-10 (anti-inflammatory cytokines), and downregulation of TNF-α and IL-6 (proinflammatory cytokines) by threefold and IL-6 by eightfold (proinflammatory cytokines) were observed.  *P. acidilactici* TMAB26 demonstrated high adhesion efficiency to HT-29 cells | (Barigela and Bhukya, 2021) |
| 2. | Kallappam  (South Indian fermented food made with rice flour and coconut batter) | *Lactobacillus plantarum* AS1 | In vivo study using normal and tumour induced rats. After a feeding regime of 26 weeks, the antioxidant activity of colon tissue was measured by measurement of thiobarbituric acid reactive substances. | Kallappam-fed rats exhibited 50.96% antioxidant activity (peroxidation inhibition of linoleic acid) and 29.15% free radical scavenging activity, causing a 42.13% reduction in tumor number and a 36.12% reduction in tumor size compared to control. Enhanced antioxidant enzyme activities (SOD, CAT, GST) and decreased lipid peroxidation were observed in the AS1-treated group. | (Satish Kumar et al., 2012) |
| 3. | Panchamirtham  (A fermented fruit mix made from banana, brown sugar, seedless dates, sugar candy, honey, cardamom and ghee) | *Bacillus valezensis* M4S1B1, *Proteus. terrae* M7S2B1 313 | In vitro tests using the isolated strain were carried out. Cytotoxic assays were conducted and the adhesion of the strain on human lung cells were observed. | Showed potential activity against lung cancer cell line A549 with low IC50 values (Cellular shrinkage and membrane damage)  The adhesion study showed that the strain adhered to human lung cancer cell lines, A549, with significant cytotoxic effects. | (Uma Maheshwari et al., 2019) |
| 4. | Dasamoolarishta  (Indian Ayurvedic fermented traditional medicine) | *Bacillus licheniformis* AG-06, *Bacillus albus* DM-15 | The probiotic bacterial strain, DM-15, was isolated from Dasamoolarishtam and in-vitro anti-proliferation tests were conducted using A549 cancer cell line | The strain exhibited antiproliferative activity, with significant cytotoxicity observed at 20μg/mL against A549 cancer cell line. | (Vinothkanna et al., 2022) |
| 5. | Fermented banana (*Musa*  *Paradisiaca*) | *Lactobacillus casei, Bifidobacterium bifidum* | In vitro study in which the cytotoxic effects of the fermentation supernatant of *Musa paradisiaca* dietary fiber on HT29 cell lines were assessed using MTT release assay. | *B. bifidum* produces important SCFAs such as butyrate which exhibits anti-cancer properties  The inhibition of β-glucuronidase (an enzyme involved in the hydrolysis of glucuronides that can lead to the reactivation of carcinogenic compounds in the colon) by the fermentation supernatant from PIF was 53.78% for *B. bifidum* and 42.36% for *L. casei*.  MTT Assay showed cytotoxicity of the fermentation supernatants, with *B. bifidum* being more effective than *L. casei*. | (Arun et al., 2019) |
| 6. | Dahi  (Indian fermented milk product) | *Lactobacillus acidophilus, Bifidobacterium bifidum* | In vivo study involving normal rats and cancer induced rats using the carcinogen, 1, 2-dimethyl hydrazine (DMH). Rats were divided into 7 groups and each group was given different compositions of dietary treatment which included either just the probiotic dahi or a combination of dahi and piroxicam (anti-cancer synthetic drugs).  Expression of PD-1 in colorectal tissues was studied by immunohistochemical staining. | Probiotic dahi showed promising anti-cancer effects against colorectal cancer.  Probiotic dahi or piroxicam individually reduced PD-1 expression in DMH-induced colorectal mucosa. However, their combined treatment showed significantly greater efficacy in diminishing PD-1 expression.  The combination of piroxicam and probiotic Dahi exhibited a synergistic effect, inhibiting the initiation and progression of neoplastic lesions. | (Mohania et al., 2013) |
| 7. | Tomato Pickle | *Weissella cibaria* p3B | In vitro study where anti-proliferative effect of the isolated strain was tested using MTT assay. | Showed antiproliferative properties against the HeLa cell line, with the IC and CE of *W. cibaria* p3B exhibiting the highest activities (64.54% and 50.23%, respectively). The IC. MIX of bacterial isolates displayed even greater antiproliferative activities (77.53%) compared to the CE. MIX (55.46%) and the individual isolates. | (Ankaiah et al., 2021) |
| 8. | Utonga-kupsu  (Manipuri fermented fish product) | *Staphylococcus* sp., *S. carnosus*  *S. piscifermentans* | In vitro study where cytotoxic effects of the strain against HeLa, HT-29 and normal lung cells were tested using MTT assay. | Displayed 78% cytotoxicity against cancer cell lines but only 12% against normal cells. | (Singh et al., 2018) |
| 9. | Babru  (Pancakes made from fermented batter),  Aara, Apple wine  (Fermented drinks of North-Western Himalayas) | *Lactobacillus plantarum* strains (AdF3, AdF5, AdF6, AdF7, AdF9, AdF10), *Enterococcus faecium* strains (AdF11, AdF2), *Saccharomyces cerevisiae* strains (Sc12, Sc04, Sc17) | In vitro study where the isolated strains were assessed for their ability to inhibit genotoxins and mutagens through SOS chromotest and Ames test. Viability of the strains against 4-NQO and furazolidone, and their ability to bind and transform carcinogens were also evaluated. | The isolated strains showed high degree of survival after genotoxin exposure (with more than 80% viability), significant inhibition of genotoxins, high antimutagenicity against tested genotoxins (70%), high antigenotoxicity and over 90% inhibition of 4-NQO and 75% inhibition of furazolidone, effective binding and potential metabolic transformation of carcinogens | (Walia et al., 2014) |

**Table S2 Detailed methodology and results of studies on antidiabetic activity of Indian fermented foods**

| **S.NO** | **INDIAN FERMENTED FOOD** | **PROBIOTIC STRAIN** | **METHODOLOGY OF THE STUDY** | **ANTIDIABETIC ACTIVITY REPORTED** | **REFERENCE** |
| --- | --- | --- | --- | --- | --- |
| 1. | Fermented rice | *Bifidobacterium sp., Lactobacillus sp.* | In vivo study using rats. Rats were randomly grouped into four groups and given different diets- normal diet (ND), high fat diet (HFD), high fat diet with control food sample (HFDC), high fat diet with test sample (HFDT). After 8 weeks of feeding, biochemical analysis of serum-glucose tolerance test and insulin tolerance test, histological examination of the liver and epididymal adipose tissue were carried out. | After 120 minutes of insulin injection, the blood sugar levels stayed high in both the HFD and HFDC groups, whereas the blood sugar levels in the HFDT group stayed within the healthy range. The levels of insulin and leptin in the HFD and HFDC groups showed an approximate twofold increase compared to the ND group. On the other hand, the HFDT group exhibited a significant improvement of 77.89% in adiponectin levels when compared to the HFD group. | (Hor et al., 2022) |
| 2. | Milk-millet composite probiotic fermented product (finger millet) | *Lactobacillus helveticus* MTCC 5463 | In vitro study using the probiotic strain *Lactobacillus helveticus* MTCC 5463 isolated from fermented food. Microbiological analysis, antimicrobial analysis, antioxidant activity was evaluated using 2,2’-Azino-bis (3-ethylbenzothiazoline-6-sulfonic acid) (ABTS) assay. The anti-diabetic activity was assessed by measuring the inhibition of α-amylase and α-glucosidase. | Incorporation of finger millet with milk has improved antimicrobial, antioxidant, and anti-diabetic potential. This diet plan inhibits the production of glucose by inhibiting enzymes that break down carbohydrates, such as α-amylase and α-glucosidase. | (Chaudhary and Mudgal, 2020a) |
| 3. | Millet-legume-based Indian traditional fermented product | *Lactobacillus plantarum, Lactiplantibacillus plantarum, Enterococcus faecalis, Saccharomyces cevisiae* | In vivo study using streptozotocin-induced diabetic rats. The rats were separated into four distinct groups and were provided with varying diets. Group 1 consisted of normal rats that were fed a basal diet, while group 2 comprised of normal rats that were fed an experimental diet. Group 3 consisted of diabetic rats that were fed a basal control diet (DC), and group 4 comprised diabetic rats that were fed an experimental diet (DE). After 4 weeks of feeding, biochemical analysis of serum, glucose and glycosylated Hb levels and histopathological analysis of kidney tissue were conducted. | Incorporating whole finger millet and seed coat matter into the diet resulted in a reduction in plasma glucose levels in diabetic rats. Histopathological analysis showed that the glomerulus and tubule structure in the NC group appeared normal. In contrast, the DC group displayed a shrunken glomerulus with thickening in the mesangial cells. On the other hand, the DE group exhibited a normal glomerulus with minimal thickening in the mesangial cells, indicating the anti-nephropathic effect of the fermented food. | (Devi and Rajendran, 2023) |
| 4. | Sprouted and fermented quinoa | Not mentioned | In vivo study using rats. Rats were divided into 6 groups, with major groups being- consisting of a standard control diet, a control diet with a high glycemic index, and diets with a high glycemic index along with supplementation of processed quinoa flour. After 47 days of feeding, postprandial blood glucose levels, glycemic index, blood glucose levels, total cholesterol, glycated haemoglobin, triglycerides, and cholesterol fractions were measured. | The inclusion of quinoa flour in the diet resulted in a decrease in the glycemic index of the diet, food consumption, blood glucose and lipid levels, as well as the accumulation of epididymal adipose tissue. | (Lopes et al., 2019) |
| 5. | Fermented amla beverage  (Gooseberry) | *Pediococcus lolii, Lactobacillus plantarum, Pediococcus. acidilacti, Pediococcus pennaceous* | In vivo study using streptozotocin induced diabetic rats. The rats were separated into five groups: the normal control group (received only food and water), the diabetic untreated group (received food and water), the diabetic standard group (administered 2.0 mg/kg/day of the hypoglycemic drug, glibenclamide orally), the diabetic experimental group 1 (given 2 ml/kg of non-fermented Amla beverage orally starting from the day of diabetes induction), and the diabetic experimental group 2 (given 2 ml/kg of fermented Amla beverage orally).Measurement of fasting blood glucose, body weight, and histological analysis using liver tissue were conducted. | Histopathological studies of Amla fed rats showed reduced liver damage. The hepatocytes had no fat vesicles or inflammation, and the cellular structure remained intact.  Diabetic experimental animals that were given FAB (fermented amla beverage) showed a significant reduction in fasting blood glucose levels compared to the diabetic standard group, which received the hypoglycemic drug glibenclamide, as well as the untreated diabetic animals. | (Modi et al., 2023) |
| 6. | Finger millet -enriched probiotic fermented milk | *Lactobacillus helveticus* MTCC 5463 | In vivo study using rats. Rats were divided into six groups and fed different diets- including two control groups (one comprising of non-diabetic rats and one comprising of diabetic rats), and four groups of diabetic rats fed with different diets such as - probiotic fermented milk, finger millet-enriched probiotic fermented milk, finger millet flour, or metformin (which is the standard drug). After 4 weeks of feeding, biochemical analysis-blood glucose, triglycerides, histopathological analysis of liver and pancreatic tissue. | The consumption of metformin, finger millet-enriched milk, and finger millet flour by the groups resulted in reduced blood glucose levels. Histopathology study showed that the administration of probiotic milk enriched with millet, finger millet flour, and probiotic fermented milk partially improved the inflammation and changes in liver structure  The group that consumed the probiotic milk fortified with finger millet showed a reversal in the activities of hepatic marker enzymes such as alanine transaminase (ALT) and aspartate transaminase (AST). (Elevated levels of both ALT and AST are indicative of liver disease and are commonly found in people with diabetes). | (Chaudhary and Mudgal, 2020b) |
| 7. | Dahi (fermented milk product) | *Lactobacillus acidophilus, Lactobacillus lactis, Lactobacillus casei* | In vivo study using male albino Wistar rats. Rats were divided into 3 groups- normal control group (standard diet), high fructose-fed control group (standard diet +21% fructose solution), dahi- and high fructose-treated group (a standard diet +15% dahi and 21% fructose). glycosylated haemoglobin, fasting blood glucose, oral glucose tolerance test, liver glycogen content, plasma insulin, and blood lipid profile were recorded. | HbA1c levels and fasting blood glucose in the HFCG (high fructose-fed control group) group were significantly higher (53%).  Dahi displayed low GI index, which prevents or delays the onset of disease. Dahi displayed antioxidative activity and delayed glucose intolerance, reduced hyperinsulinemia, hyperglycaemia, dyslipidemia, and oxidative stress in diabetic rats which indicates a lower risk of diabetes. | (Yadav et al., 2007) |
| 8. | Fermented papaya | *Levilactobacillus brevis* RAMULAB52 | In vitro study using probiotic strain isolated from fermented papaya and its cell free supernatant. Probiotic strain was evaluated for its inhibitory activity of α-amylase enzymes and α-glucosidase, antibacterial activity, and antioxidant activity. In vitro adhesion to chicken crop epithelial cells, HT-29 cells and buccal epithelial cells was also evaluated. | Cell free supernatant of the probiotic strain demonstrated 86.97% and 75.87% inhibition of the activities of α-glucosidase and α-amylase, respectively. Cell free supernatant also exhibited anti-bacterial and anti-oxidant activity.  *L. brevis* strain showed adhesion to different cells such as buccal epithelial cells, HT-29 cells and chicken crop epithelial cells. | (Sreepathi et al., 2023) |
| 9. | Skim Milk and Dahi (Yogurt) | *Lactococcus lactis ssp. lactis, L. lactis ssp. cremoris, L. lactis ssp. diacetylactis, and Leuconostoc citrovorum* | In vivo study using rats. Three groups of rats were provided with varying diets: a control group (fed with a high-fructose diet), a group fed with skim milk (supplemented with a high-fructose diet), and a group fed with dahi (supplemented with a high-fructose diet). After 42 days, the rats were examined for oral glucose tolerance test, HbA1c, blood glucose, and insulin levels. | The animals fed with dahi and skim milk had significantly lower blood glucose levels compared to the control animals, with reductions of 10% and 17% respectively. Additionally, their HbA1c levels were also significantly lower, with reductions of 10% and 17% respectively. The glucose levels for the diet groups supplemented with skim milk and dahi were markedly lower compared to the control group. Plasma insulin levels were notably reduced in animals fed with skim milk and dahi, showing a decrease of 34% and 48% respectively, compared to the control group. | (Yadav et al., 2006) |
| 10. | Dosa batter | *Limosilactobacillus fermentum, Lactisaseibacillus casei* | In vitro study using probiotic strains isolated from dosa batter and its cell free supernatant. Gastrointestinal tolerance, adherence,  hydrophobicity, antioxidant activity, antimicrobial activity were estimated.  The inhibitory potential of the cell-free extract (CE), cell-free supernatant (CS), and intact cells (IC) was assessed against the carbohydrate hydrolysing enzymes α-amylase and α-glucosidase. | The inhibition of α-glucosidase ranged from 7.50% to 65.01% for CS, CE, and IC, while the inhibition of α-amylase ranged from 20.21% to 56.91%. Intact cells showed the least amount of inhibition when compared to the supernatant and pellets out of the three samples. Each of the isolates displayed promising antimicrobial activity against *M. luteus* and *P. aeruginosa*. The isolates displayed a wide range of scavenging activity for ABTS radicals, varying from 20.77% to 89.75%. Among them, RAMULAB07 exhibited the highest scavenging activity at a concentration of 10^3^ CFU/mL cells. Each of the six isolates demonstrated optimal growth in gastrointestinal juice. | (Kumari V. B. et al., 2022) |
| 11. | Fermented buffalo and camel Milk | *Limosilactobacillus fermentum* (KGL4), *Saccharomyces cerevisiae* (WBS2A) | In vitro study using probiotic strain isolated from fermented food. Isolation and identification of the strain were carried out. Anti-diabetic activity and ACE inhibitory activity were analyzed using the strain. | In comparison to fermented buffalo milk (FBM), fermented camel milk had the highest levels of alpha-glucosidase inhibitory, lipase inhibitory, and alpha-amylase inhibitory activities (77.96 ± 2.61, 85.37 ± 2.15, and 70.86 ± 1.02). | (Khakhariya et al., 2023) |
| 12. | Fermented jalebi, medhu vada, and kallappam batters | *Lacticaseibacillus rhamnosus* RAMULAB13, *Lactiplantibacillus plantarum* RAMULAB14, *Lactiplantibacillus pentosus* RAMULAB15, *Lacticaseibacillus paracasei* RAMULAB16, *Lacticaseibacillus casei* RAMULAB17, *Lacticaseibacillus casei* RAMULAB20, *Lacticaseibacillus paracasei* RAMULAB21 | In vitro study using probiotic strain isolated from fermented food. Seven strains were isolated in this study. Inhibitory assay (α-amylase and α-glucosidase), antimicrobial activity and antioxidant activity were analysed using the strains. | The cell-free extract, cell-free supernatant, and intact cells of the isolates exhibited α-glucosidase inhibition ranging from 15.08 to 59.55%, while α-amylase inhibition varied between 18.79 and 63.42%. The strain RAMULAB15 demonstrated the highest inhibition rate for both α-glucosidase and α-amylase, with values of 59.55 and 63.42%, respectively. RAMULAB15 showed highest (76.63%) radical scavenging activity. RAMULAB15 demonstrated significant antimicrobial effectiveness against every pathogen tested. | (Huligere et al., 2023) |

**Table S3 Detailed methodology and results of studies on anticholesterolemic activity of Indian fermented foods**

| **S. No.** | **INDIAN FERMENTED FOOD** | **PROBIOTIC STRAIN** | **METHODOLOGY OF THE STUDY** | **ANTICHOLESTEROLEMIC ACTIVITY REPORTED** | **REFERENCE** |
| --- | --- | --- | --- | --- | --- |
| 1. | Fermented rice | *Bifidobacterium* sp. MKK4 | In vivo study using rats. The rats were randomly assigned into 4 groups: one with a normal diet, another with a high fat diet (HFD), a third with HFD supplemented with *Bifidobacterium* sp. MKK4 (HFDB), and the last one with HFD supplemented with MKK4 associated rice-fermented food (HFDBF). After 8 weeks of feeding, biochemical analysis of serum, analysis of lipid profile, anti-obesity activity, and analysis of liver functions were carried out. | Analysis of lipid profile of the HFDB and HFDBF group showed reduction in cholesterol levels, with a decrease of 53% and 56% respectively.  *Bifidobacterium* sp. MKK4 exhibited BSH activity and was found to possess the BSH gene which was responsible for their anti-obesity effect. The functional analysis of liver shows a significant elevation in the SGPT (serum glutamic-pyruvic transaminase) and SGOT (serum glutamic- oxaloacetic transaminase) levels in mice fed with a HFD, while normal levels were observed in mice from the HFDB and HFDBF groups. | (Ray et al., 2018) |
| 2. | Fermented koozh (rice-based food) and gherkin (fermented cucumber) | *Weissella koreensis* FKI21, *Lactobacillus crispatus* GI9 | In vitro study using probiotic strains isolated from fermented food. The isolated strains were identified and phylogenetic analysis was carried out from the fermented food. Survival under stimulated conditions, resistance to bile concentrations, cholesterol reduction activity, and deconjugation of sodium taurocholate and sodium glycocholate were analyzed using the isolated strains. | After 3 hours of experimental period, *W. koreensis* FKI21 and *L. crispatus* GI9 showed increased resistance to all pH levels. They exhibited enhanced tolerance to bile salts and thrived in environments with low pH levels.  All the isolated strains demonstrated a notable decrease in cholesterol levels when the media was enriched with 50 mg/ml of cholesterol.  *W. koreensis* FKI21 and *L. crispatus* GI9 showed cholesterol-reducing properties. The strains were recognized for their ability to release higher levels of cholic acid, deconjugate sodium glycocholate and sodium taurocholate, and absorb a greater amount of cholesterol from the growth medium. | (Anandharaj et al., 2015) |
| 3. | Kalarei  (Fermented milk product) | *Enterococcus faecium* K2 | In vitro study using probiotic strain isolated from fermented food. The strain was assessed for its ability to deconjugate bile, adsorb cholesterol, lower cholesterol levels, and assimilate cholesterol. | The K2 isolate showed robust deconjugation capabilities when cultured in MRS broth. Probiotic strain K2 exhibited a notable capacity to attach cholesterol to its cellular membrane when compared to the control group. It exhibited in vitro cholesterol reduction capability of 82.32%. | (Bhat et al., 2019) |
| 4. | Yogurt | *Enterococcus faecium* Chloe1, *Enterococcus faecium* EF, *Lactobacillus lus pentosus* 7MP | In vitro study using probiotic strains isolated from fermented food. Isolation and identification of LAB from yogurt followed by evaluation of   cholesterol reduction activity, and bile salt hydrolase activity. | The 7MP isolate exhibited the highest BSH activity among all the isolates. The 7MP isolate exhibited the highest potential for reducing cholesterol (48%) when compared to the other isolates. The cholesterol reduction levels achieved by each of the three isolates varied from 42% to 48%. | (Walhe et al., 2021) |
| 5. | Fermented Indian food | *Pediococcus pentosaceus* G11, *Pediococcus pentosaceus* AP3 | In vitro study using probiotic strains isolated from fermented food. LAB isolates were identified and observed for bile salt hydrolase activity. Cholesterol reduction activity was evaluated by enzymatic method and growing the cultures in MRS broth containing cholesterol. | The BSH activity was evidenced by the zone of precipitation observed in the LAB cultures. It was observed that all LAB isolates decreased cholesterol levels in the broth. Strain AP3 showed 50% decrease in cholesterol levels, while G11 showed 40% reduction. | (Rajan et al., 2021) |
| 6. | Yogurt | *Bifidobacterium bifidum* 2715 | In vivo study using rats divided into 9 groups: basal diet (Group 1), basal + cholesterol (Group 2), basal + cholesterol + yogurt A1 (Group 3), basal + cholesterol + bifidus yogurt B1 (Group 4), basal + cholesterol + yogurt A2 (Group 5), basal + cholesterol + bifidus yogurt B2 (Group 6), basal + cholesterol + yogurt A3 (Group 7), basal + cholesterol + bifidus yogurt B3 (Group 8), basal + cholesterol + whole milk (Group 9).  A1, A2, A3, yogurts prepared with *Streptococcus thermophilus* and *Lactobacillus delbrueckii* subsp. *bulgaricus*; B1, B2, B3, yogurts prepared with *Streptococcus thermophilus*, *Lactobacillus delbrueckii* subsp. *bulgaricus* and *Bifidobacterium bifidum*; A1 and B1 yogurts were fortified with skim milk powder; A2 and B2 yogurts were fortified with condensed whey; A3 and B3 yogurts were fortified with lactose-hydrolysed condensed whey.  Basal diet consists of ground nut, sesame, wheat, wheat bran, mineral mix and vitamin mix. After 30 days of feeding, the rats were analyzed for serum triacylglycerol, HDL, total cholesterol, and LDL. Cholesterol was given at 5 g/kg body weight. | A notable reduction in total cholesterol levels was observed in both A3 and all Bifidus yogurts. The lactose-hydrolyzed condensed whey proved to be the most efficient in lowering serum cholesterol levels, with condensed whey and skim milk powder following closely behind. Elevated levels of serum triacylglycerols were observed in all groups that consumed yogurt in comparison to the milk group. Rats that were administered yogurts A2 and A3 exhibited lower LDL levels compared to rats that received the milk supplement. Yogurt B1 group shows rise in HDL level. | (Athrayilkkalathil et al., 1997) |
| 7. | Probiotic dahi  (Fermented milk product) | *Lactobacillus fermentum* strains, i.e., PH5 (handva batter isolate) and PD2 (dosa batter isolate). | An in vivo study was conducted using rats that were divided into 7 groups: group A received a normal diet consisting of regular standard diet pellets, group B received a model diet which was an atherogenic diet (hyperlipidemic diet), group C received a standard diet along with an atherogenic diet and a standard anti-hyperlipidemic drug. Groups D, E, F, and G received an experimental diet which included an atherogenic diet along with dahi containing probiotic strains PD2 (10^7^ cfu/ml), PD2 (10^9^ cfu/ml), PH5 (10^7^ cfu/ml), and PH5 (10^9^ cfu/ml), respectively. After 4 weeks of feeding, the rats were assessed for serum cholesterol level, LDL and HDL levels, as well as liver cholesterol level. Furthermore, histopathological analysis of the rat liver tissue was conducted. | Group G displayed the highest potential for lowering total cholesterol. Atorvastatin given to group C (Standard) resulted in a 51.95% increase in serum HDL levels, the highest among all groups, in comparison to group B (Model). The maximum LDL level decreased to 101.72 mg/dl in Group G. The administration of PD test doses 1 and 2 (Group D and E) resulted in a notable decrease in liver cholesterol levels by 24.97% and 16.34%, respectively.   Histopathological examination of liver tissue revealed that Group A (Normal) exhibited the lowest level of liver triglycerides. Conversely, group B (Model) displayed a moderate degree of vacuolization and an elevation in lipid deposition within the cytoplasm. The inclusion of probiotic dahi containing PD2 and PH5 strains notably mitigated hepatocyte steatosis in comparison to group B (Model). | (Thakkar et al., 2020) |
| 8. | Fermented cereal-pulse food mixture | *Lactobacillus casei* (NCDC-19), *Saccharomyces boulardii* | An in vivo experiment was conducted using rats that were separated into two groups: one group was fed a control diet consisting of unfermented food mixture with additional 1% cholesterol, while the other group was fed an experimental diet consisting of fermented (*S. boulardii + L. casei*) food mixture with additional 1% cholesterol. After 42 days of feeding, the serum lipid profile (HDL, LDL, triglycerides levels) and liver cholesterol level were assessed. | The experimental group showed a significant 19% decrease in overall serum cholesterol levels as a result of consuming the fermented food blend. The experimental group showed 11% increase in HDL cholesterol, while the control group only saw 3% increase. The experimental group experienced 9% decrease in triglycerides, while the control group saw 3% reduction. A 37% decline in LDL level in the experimental group was observed. The experimental group had a liver cholesterol level of 0.08 g/100 g of tissue, whereas the control group had a liver cholesterol level of 0.12 g/100 g of liver tissue. | (Sindhu and Khetarpaul, 2003) |
| 9. | Fermented milk | *Lactobacillus rhamnosus* 5957, *Lactobacillus rhamnosus* 5897 | In vivo study involving rats divided into 5 groups- supplemented with standard diet, high cholesterol diet (HCD), HCD + milk group, HCD + *L. rhamnosus* 5957 group-fermented milk, and HCD + *L. rhamnosus* 5897 group-fermented milk. After 30 months of feeding, lipid profile, antioxidant activity, and faecal microbial counts were analyzed. | Lipid profiling revealed that serum triglycerides, total cholesterol, and LDL were significantly lower in the (probiotic fermented milk) PFM-fed groups.  Catalase activity was significantly enhanced by the probiotic strains LR 5957 and LR 5897, indicating strong antioxidant effects. However, only LR 5957 exhibited a significant 1.3-fold increase in superoxide dismutase activity compared to the HCD group. There was no statistically significant increase in glutathione peroxidase activity in the milk and probiotic fermented milk (PFM).  The faecal analysis revealed a 1.35-fold increase in colony-forming units on MRS in both PFM groups (LR 5957 and LR 5897) and a 1.2-fold increase in the milk group compared to the HCD-fed group, leading to restoration in different treatment groups. | (Yadav et al., 2019) |

**Table S4 Detailed methodology and results of studies on antibacterial activity of Indian fermented foods**

| **S. No.** | **INDIAN FERMENTED FOOD** | **PROBIOTIC STRAIN** | **METHODOLOGY OF THE STUDY** | **ANTIMICROBIAL PROPERTY REPORTED** | **REFERENCE** |
| --- | --- | --- | --- | --- | --- |
| 1. | Tungtap (fermented ﬁsh) and Tungrymbai (fermented soybean) | *Lactobacillus plantarum, Lactobacillus brevis, Lactobacillus casei, Lactobacillus fermentum, Pediococcus pentosaceus, Enterococcus sp.* | In vitro study using the cell-free supernatants (CFS) derived from the LAB strains isolated from fermented foods. Antimicrobial activity against nosocomial bacteria that produce lactamase, both individually and in combination with antibiotics like cefotaxime, imipenem, tigecycline, and polymyxin B was evaluated using the agar spot assay test. | The bacteriocin extracts had a minimum inhibitory concentration (MIC) ranging from 6.66 to 26.66 mg/ml for *Pediococcus pentosaceus* LU11 and from 10 to 33.33 mg/ml for *Lactobacillus plantarum* LS6. | (Biswas et al., 2017) |
| 2. | Dosa batter (fermented cereal-pulse based food) | *Lactobacillus plantarum* LD4 | In-vitro study using the CFS obtained from an overnight culture of *Lactobacillus plantarum* strain LD4. CFS was used for the bacteriocin activity assay. The mode of action and host range of the bacteriocin were investigated. | *Salmonella typhi, Micrococcus luteus* and a few species of LAB were among the Gram-positive and Gram-negative bacteria against which antimicrobial action was reported.  In target cells, bacteriocin LD4 induced K^+^ ion efflux, indicating that the substance is pore-forming. | (Kumar et al., 2016) |
| 3. | Uttappam batter, dosa batter  (fermented cereal-pulse based food) | *Lactobacillus rhamnosus, Lactobacillus plantarum, Lactobacillus brevis* | In vitro study using CFS of the isolated cultures. The anti-microbial compound in the CFS was identified by ESI-MS and fragmentation pattern analysis. The agar well diffusion method was used to assess the antibacterial activity of the purified compound against different organisms. | The low molecular weight compound was identified as 2-hydroxyl indole-3-propanamide from ESI-MS.  It showed antibacterial action, against both Gram-positive and Gram-negative pathogenic bacteria during agar well diffusion assay. | (Jeevaratnam et al., 2015) |
| 4. | Hentak  (traditional fermented fish product) | *Lactobacillus pentosus* | In vitro study using CFNS of the isolated strain. Antibacterial activity of the CFNS was evaluated using the agar well diffusion method.  The CFNS was evaluated for potential antioxidant properties through DPPH(1,1-diphenyl-2-picrylhydrazyl) free radical scavenging activity. | This strain was found to produce antibacterial substances that showed growth inhibitory activity against several human enteric pathogens, including *Staphylococcus epidermidis*, *Micrococcus luteus*, *Shigella flexneri*, *Yersinia enterocolitica*, and *Proteus vulgaris*.  The CFNS exhibited significant DPPH free radical scavenging activity in a dose-dependent manner, ranging from 8.8 ± 0.12% to 57.35 ± 0.1% at concentrations from 100 to 1000 μl. | (Aarti et al., 2016) |
| 5. | Kinema (fermented soybean food),  Dahi (fermented milk product) | *Lactobacillus plantarum* DHCU70, *Lactobacillus plantarum* DKP1 | In vitro study using the CFS of the isolated strains. The antibacterial effectiveness was assessed by using CFS in the agar well diffusion assay, targeting the *K. rhizophila* indicator strain. Sequence similarity searches using BLASTP was used to identify the bacteriocin encoding genes and probiotic functionalities. | *The strains* DHCU70 and DKP1 of Lactobacillus plantarum exhibited strong antimicrobial effects against the indicator strain *Kocuria rhizophila* ATCC 9341*.* Whole cell reporter assay was used to determine that the bacteriocin produced by the *L. plantarum* strains DHCU70 and DKP1 acted by inhibiting cell wall biosynthesis, but did not affect DNA or fatty acid biosynthesis. The genomic analysis of L. plantarum strains DHCU70 and DKP1 revealed a genetic basis for bacteriocin production. The bacteriocin encoding locus (pln locus) in both strains consists of 23 genes, including two-peptide bacteriocins and inducible class II plantaricin NC8αβ. Both strains have a regulatory operon and a two-component signal transduction system. | (Goel et al., 2020) |
| 6. | Shidal  (salted fermented fish food) | *Lactobacillus plantarum* LA21 | In vitro study using cell-free extract of *L. plantarum* LA21 to assess its antibacterial activity against *Bacillus pumilus*, *Staphylococcus aureus*, *Bacillus amyloliquefaciens*, and *Listeria monocytogenes* by employing the agar well diffusion method. | The pathogens *Bacillus pumilus*, *Staphylococcus aureus*, *Bacillus amyloliquefaciens*, and *Listeria monocytogenes* were susceptible to the cell-free extract. MIC value was noted for *L. monocytogenes*.  Molecular docking studies demonstrated strong interactions between the bacteriocin and the target pathogens' surface proteins and transcriptional regulator proteins. | (Leslie et al., 2021) |
